# Co-ViTDefect: A Bayesian Ensemble of Vision Transformers for Multiclass Concrete Defect Identification with Interpretability

**DOI:** 10.3390/ma19214591

**Published:** 2026-10-29

**Authors:** Nizar Faisal Alkayem, Ali Mayya, Xin Zhang, Lei Shen, Na Zhang, Mahmoud Bayat, Qiang Wang, Maosen Cao

**Affiliations:** 1 College of Automation, Nanjing University of Posts and Telecommunications, Nanjing 210046, China; 2 Innovative Technology Department, MeDiTech Institute, University of Art and Applied Science of Southern Switzerland, 6900 Lugano, Switzerland; 3 College of Water Conservancy and Hydropower Engineering, Hohai University, Nanjing 210098, China; 4 School of Architecture, College of Architecture, Planning and Public Affairs, The University of Texas at Arlington, Arlington, TX 76019, USA; 5 College of Mechanics and Engineering Science, Hohai University, Nanjing 211100, China

**Keywords:** vision transformers, ensemble learning, multiclass classification, concrete structures

## Abstract

In-service concrete structures often suffer from various types of defects. Automatically identifying multiple defect types in operating concrete structures remains challenging for inspectors and maintenance engineers worldwide. Therefore, efficient tools for evaluating surface defects in concrete structures are essential. In particular, vision transformers (ViTs) are relatively new tools for computer vision tasks and often outperform traditional convolutional neural networks (CNNs). Several attempts have utilized ViTs for crack classification in concrete structures, but they mostly rely on single-model identification and implement binary classification. By leveraging ensemble learning, this work proposes a novel ensemble approach, Co-ViTDefect, for multiclass concrete defect classification. The model integrates multiple ViT learners using Bayesian Dirichlet-based ensemble learning, which probabilistically balances the strengths of individual models and also provides an estimate of the uncertainty of each prediction. The model was evaluated with five-fold cross-validation. Experimental results show that the proposed Co-ViTDefect achieves 88.19% accuracy and 85.1% F1-score, with per-class F1-scores above 90% for cracks, efflorescence, and no-defect categories. It outperformed the best single ViT model (87.48% accuracy, 84.16% macro F1-score) as well as CNN-based and transformer-based models, including Swin-Tiny and MaxViT-Tiny. Without any post hoc calibration, Co-ViTDefect also had a lower calibration error than the uniform averaging ensemble (ECE of 0.214 against 0.285) and the lowest Brier score (0.242). Future work can improve the recognition of scaling and general defects.

## 1. Introduction

Concrete structures are critical transportation infrastructure that strongly influence the smooth flow of people and goods across natural barriers [1,2]. Nevertheless, these structures often face severe environmental conditions, such as overloading, material degradation, and earthquakes, that cause various defects [3,4]. If left undetected and unaddressed, these defects threaten safe operation and structural integrity, potentially leading to catastrophic failures with devastating economic and human losses [5,6]. Hence, health monitoring and early defect identification in concrete structures are crucial for real-time inspection, rapid intervention, and preventing tragic events [7,8].

Concrete structures exhibit various defects, including cracks, scaling due to deterioration of the concrete surface, delamination, spalling caused by flaking or peeling of the concrete, efflorescence from surface salt deposition, and general defects such as exposed reinforcement and other special crack types. Each defect category shows distinct visual characteristics driven by different underlying damage mechanisms. Accurate classification is necessary to define appropriate maintenance strategies and rehabilitation actions [9,10]. Professional technicians’ inspection procedures are costly, time-consuming, and laborious, and do not provide comprehensive monitoring of many in-service concrete structures [11,12].

In recent years, deep learning and machine vision technologies have advanced rapidly, improving methods for defect identification through surface visual inspection [13,14]. Among these methods, convolutional neural networks (CNNs) dominate machine vision applications and are commonly used for visual damage identification and defect diagnosis in concrete structures [15,16]. Nevertheless, CNNs may struggle to capture spatial contextual relationships efficiently because of their locally constrained receptive fields and strong spatial inductive biases. These characteristics limit their performance in multiclass defect identification under the complex, visually challenging, and noisy conditions of in-field concrete structure defect inspections [17,18].

Vision Transformers (ViTs) have emerged from natural language processing as powerful tools that adapt to image data via self-attention and have been introduced to machine vision [19]. ViTs are efficient, intelligent tools that are being gradually utilized for crack classification [20]. Unlike CNNs, which are limited by the hierarchical architecture and locally constrained receptive fields, ViTs can identify long-range dependencies across the entire input image using self-attention. Hence, ViTs capture the local and global features required in complex concrete structure surface images [21]. By targeting relevant pixel regions across an input image, ViTs process the image at various scales, which helps identify multiple defect types in concrete structures, a task some CNN-based approaches may fail to tackle. Furthermore, the self-attention mechanism lets ViTs focus on different regions of the concrete structure image, depending on the multiclass defect identification task [22,23].

While single ViT models have achieved outstanding results across various machine vision tasks, recent progress in ensemble learning shows that combining multiple ViTs can further improve performance and robustness [24]. By exploiting the diversity in inductive biases and learned representations across different ViTs, ensemble ViTs can deliver more precise, reliable outcomes [25]. Ensemble learning combines multiple ViT models synergistically, addressing limitations of individual models, such as overfitting or sensitivity to specific environmental characteristics. Moreover, an ensemble of ViTs can enhance overall model interpretability by leveraging the complementary features of different ViT models. In this regard, ensemble ViTs can learn a broader perspective on complex defect image patterns, enabling better classification across defect types and scenarios. These ensemble models can be implemented using weighted averaging, model-voting schemes, or more advanced approaches such as stacking or boosting. By implementing and comparing these ensemble learning paradigms, the ensemble ViTs prove to be practical tools for enhancing defect detection and classification in concrete structures. Therefore, this research examines and analyzes the use of ensemble ViT architecture for the multiclass classification of concrete defects in concrete structures. A robust ensemble learning model called the Co-ViTDefect is proposed for robust multiclass concrete defect classification by combining the complementary strengths of several individual ViTs, which can assist efficient SHM and predictive maintenance planning of in-service concrete infrastructure.

## 2. Related Work, Limitations, and Contributions

In recent years, researchers have applied ViT and its variants to identify structural surface defects. For example, Shamsabadi et al. [26] used ViT to detect cracks in masonry images. The results showed that the ViT-based model outperformed the CNN-based model in accuracy, generalization, and robustness to noise. In another work, Li et al. [27] introduced a new ViT model for semantic segmentation of concrete and asphalt surfaces. The ViT model addressed the challenges of diverse structural surfaces and narrow cracks by using a hierarchical transformer architecture and a combination of CE and Dice loss functions. Experimental results show that SegFormer outperforms traditional CNN-based models, improving accuracy and efficiency in crack detection.

Other works, such as Yadav et al. [28], developed a Dual Stream Transformer Network (DSTNet)to identify cracks in building structures. DSTNet combined the strengths of CNNs and ViTs to extract local and global features, and the results showed that DSTNet outperformed traditional CNN-based methods in accuracy and efficiency for crack detection in building structures. Wang and Su [29] proposed SegCrack for pixel-level crack segmentation in concrete structures. They used a hierarchical ViT encoder to capture multi-scale features and a top–down pathway to refine them. SegCrack showed outstanding performance compared with other methods when trained on large datasets. In another work by Shamsabadi et al. [30], they proposed a ViT-based framework for crack detection on asphalt and concrete surfaces. The proposed TransUNet model, which combines a CNN-ViT backbone with an encoder–decoder architecture, outperforms traditional CNN-based models like DeepLabv3+ and U-Net in terms of accuracy and robustness to noise.

Other advanced works, such as Ma et al. [31], propose TFCF-Net, a new network architecture for pavement crack detection that combines ViTs and CNNs to effectively capture global and local features. The authors utilized Information Complementary Fusion (ICF), multi-dimensional attention (MA), and multi-dimensional attention feature representation (MAFR) modules to enhance feature extraction. Experiments on three public datasets showed that the proposed model outperformed other pavement crack detection models. Zhang et al. [32] introduced a new crack segmentation method that leverages scene information to improve accuracy, using a cascaded ViT-CNN model to extract semantic information about cracks and background objects. Experimental results on steel-concrete structures and concrete buildings demonstrated the method’s effectiveness, outperforming single ViT networks such as SegFormer.

Additionally, Wang et al. [33] proposed SwinCrack, a Swin-Transformer-based network for pavement crack detection. SwinCrack addressed the limitations of CNN-based methods by capturing long-range interactions and adaptively aggregating spatial information. Further enhancements were added to improve performance, enabling the proposed model to outperform similar models when tested on six public datasets. Lin et al. [34] introduced a pavement anomaly detection method using ViTs and self-supervised learning, reducing reliance on large, labeled datasets and achieving excellent results. Zhang et al. [35] proposed UTCD-Net for pixel-level crack detection by combining the strengths of ViTs and CNNs: ViTs extract global context features for long-range crack detection, while CNNs capture local features for thin crack detection. It was reported that by testing a dam dataset and three public crack datasets, the model demonstrated superior performance compared with other methods.

In addition, Lu et al. [36] developed an automated method for detecting dangerous work zones for crawler cranes using the Mask ViT model with a Swin Transformer backbone to recognize and segment UAV-captured images. The proposed approach was successfully applied in a real-world engineering project, yielding high effectiveness. Xiang et al. [37] developed a dual-encoder network, DTrCNet, that fuses ViTs and CNNs to improve crack segmentation performance. Experiments on the Crack3238 dataset showed that DTrCNet outperformed other segmentation models in precision, recall, F1-score, and intersection-over-union. Ding et al. [38] developed a UAV-based method for detecting and quantifying concrete cracks without reference markers using the independent boundary refinement transformer (IBR-Former). The authors demonstrated the framework’s effectiveness and practicality through UAV field detection.

Other research, such as Chu et al. [39], proposed Cascade CATransUNet, a ViT-based architecture for high-resolution crack image segmentation. The experimental results showed that Cascade CATransUNet outperformed state-of-the-art methods. The method was also successfully applied to UAV-based concrete structure crack inspection. Wang et al. [40] proposed a dual-path network that combined CNN and ViT encoders for pavement crack segmentation. The authors tested the model on three public datasets and demonstrated its superior performance compared with state-of-the-art methods. Chen et al. [41] proposed PoolingCrack, a novel, efficient ViT-based model for crack detection in civil infrastructure. PoolingCrack outperformed other models in accuracy, noise robustness, and computational efficiency. Wang et al. [42] proposed CGTr-Net, a weakly supervised hybrid network with multi-attention that combines the strengths of CNNs and Gated Axial ViT to extract local and global features. By testing on two datasets, the authors showed CGTr-Net’s excellent performance compared with other approaches.

Other representative works on pixel-level identification were conducted by Xiao et al. [43], who introduced CrackFormer, a novel hybrid-window attentive ViT framework with several boosting mechanisms for pavement crack detection. The authors tested the model on seven pavement crack detection datasets and demonstrated CrackFormer’s merit using precision, recall, and F1-score. Liu et al. [44] proposed an improved network for pavement crack segmentation, CrackFormer-II. It used a SegNet-like structure with novel ViT encoder modules that capture long-range dependencies and semantic features. CrackFormer-II was more efficient than previous versions, with faster inference and fewer parameters.

Yu and Zhou [45] proposed an improved YOLOv5 network that combines CNN and ViT (YOLOv5-CBoT) for pavement crack detection. The C2f module was introduced to optimize the network and improve gradient flow. YOLOv5-CBoT achieved competitive results on the RDD2020 dataset, outperforming other models in both accuracy and inference speed. Xiang et al. [46] proposed a ViT-based network for pavement crack detection. The developed model can be easily integrated with sensor data for industrial applications. The network was trained on datasets from the Czech Republic, Japan, and India and achieved high F1-scores on online test sets with fewer model parameters. Wu et al. [47] proposed a U-shaped CNN called TMCrack-Net designed for mural crack detection. TMCrack-Net achieved prominent performance on the Tang Dynasty tomb chamber mural dataset and Crack500.

Ju et al. [48] proposed TransMF for crack detection, addressing the challenges of complex, long crack shapes and noisy images. It used a hybrid of convolutional blocks and Swin ViT blocks to capture long-range dependencies. Extensive experiments demonstrated that TransMF outperformed other state-of-the-art methods on three benchmark datasets. Yang et al. [49] introduced a novel approach to automated pavement crack detection using a multi-scale feature fusion network based on the ViT architecture. The proposed method achieved high performance on the Crack500 and DeepCrack datasets under various lighting conditions. Al-maqtari et al. [50] evolved a thin road crack segmentation model based on an efficient multi-scale ViT with several advanced enhancement mechanisms. The model outperformed other models on the Crack11k dataset.

Quan et al. [51] proposed CrackViT, a novel ViT-based network for pixel-level crack identification. It combined CNN and ViT encoders with feature aggregation blocks to sharpen crack edges, and a multi-task supervised training strategy was employed to enhance detail accuracy, yielding excellent outcomes. Chen et al. [52] introduced LECSFormer for road crack detection, addressing challenges from shadows, diverse crack patterns, and noise. LECSFormer outperformed other methods when tested on four public datasets. Zhou et al. [53] developed MixSegNet for crack detection and segmentation, utilizing CNN and ViT modules. Comparative studies show that MixSegNet outperforms other methods on open-source datasets, achieving high precision, recall, F1-score, and mIoU. Finally, Yadav et al. [54] proposed CCTNet, which combines CNNs and ViTs to improve crack detection accuracy and efficiency, yielding superior results in both accuracy and computational efficiency for crack detection in building structures.

Multiclass defect identification can improve overall understanding of concrete structures’ conditions and support safety and reliability. ViT-based models can help overcome challenges in multiclass surface damage identification in concrete structures, including complex, multicolored backgrounds; varying illumination; diverse defect types; and imbalanced data. Based on the above literature survey, the following gaps can be identified:

- Most available studies focus on binary classification or pixel-level identification of cracks, either implemented for concrete, pavement, or roads. Nevertheless, few studies have considered multiclass identification of concrete structures.
- Most research works do not consider other types of defects other than cracks, such as scaling, spalling, efflorescence, etc.
- Although CNNs are often used for crack identification, they suffer from difficulties in efficiently capturing spatial contextual relationships within the image data due to their locally constrained receptive fields and high-level spatial inductive biases. These characteristics limit their performance in multiclass defect identification under the complex, visually challenging, and noisy conditions of in-field concrete structure defect inspections.
- To overcome such difficulties, the well-established ViT-based models are widely employed. However, most studies focus on single ViT models or their variants.

To address these problems, the significant research contributions of this work can be listed as follows:

- To solve the multiclass defect identification in concrete structures, three core ViT models are built from scratch.
- The core ViT models’ classification heads are enhanced to improve the multiclass classification task.
- Five types of concrete structure defects, namely, cracks, spalling, efflorescence, scaling, and general, besides the “no defect” class, are considered by implementing data augmentation and class-weight imbalance handling to address the data imbalance problem.
- Using ensemble learning methods with ViTs can contribute to addressing the challenges of multiclass identification of concrete structure defect images by elaborating multiple ViT-based models to boost overall robustness and performance. Hence, a novel collaborative ViT model for multiclass defect detection called the Co-ViTDefect is proposed.
- The Co-ViTDefect is built based on a weighted ensemble approach using Bayesian updating with Dirichlet ensemble learning, which can help overcome some limitations and utilize the multiple decisions of different training strategies.
- Co-ViTDefect is compared with CNN-based and transformer-based models for classification performance and with majority voting, maximum likelihood mixture, and uniform averaging for classification and probability reliability. Reliability is assessed using ECE, negative log-likelihood, and Brier score. A separate one-fold benchmark compares model-forward and fusion times for the retained ViT members and ensemble strategies. Robustness comparisons include Gaussian noise, blur, and JPEG compression.

The rest of the paper can be outlined as follows: (i) A detailed presentation of the proposed Co-ViTDefect approach, including the utilized dataset, training settings, and model development, is shown in Section 3. (ii) The results of the development of the core ViT models and the proposed Co-ViTDefect are presented in Section 4. Moreover, a comprehensive discussion of the performance of the multiclassification models and a detailed comparison are provided. (iv) Finally, concluding remarks and future scope are outlined in Section 5.

## 3. Materials and Methods

In what follows, the overall framework of the multiclass concrete structure defect identification is outlined, the exploited dataset, the implemented single ViT models, the training process and evaluation metrics, and the proposed Co-ViTDefect ensemble approach.

### 3.1. The Crack Detection Framework Using Vision Transformers

**Figure 1:**
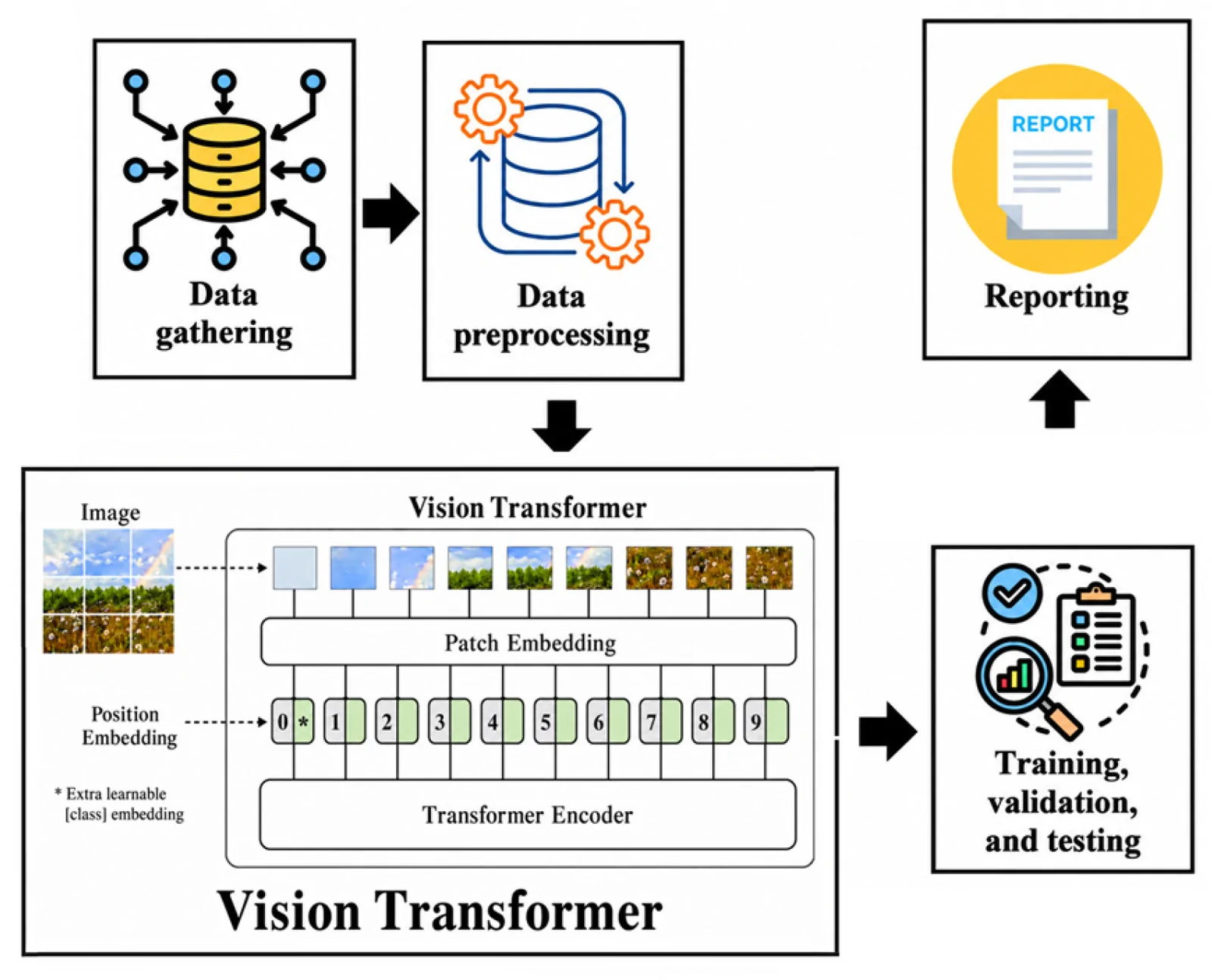
The general crack detection framework using vision transformers.

The general framework for image-based multiclass concrete structures defect identification includes four general stages, namely (i) data acquisition and preprocessing, (ii) model architecture and training algorithm, (iii) training and evaluation, and (iv) classification reporting, as depicted in Figure 1. First, raw images are captured from the concrete structures using imaging devices; then, preprocessing steps such as augmentation, cropping, normalization, noise reduction, and labeling are performed. The exploited dataset is divided into training and testing groups. A suitable ViT model and algorithm are chosen, developed, and trained until satisfactory accuracy is achieved. The trained ViT is then evaluated using the test set. Ultimately, a comprehensive report detailing the defect type is generated through an appropriate platform.

### 3.2. The Enhanced Classification Head: Vision Transformers (ViT)

**Figure 2:**
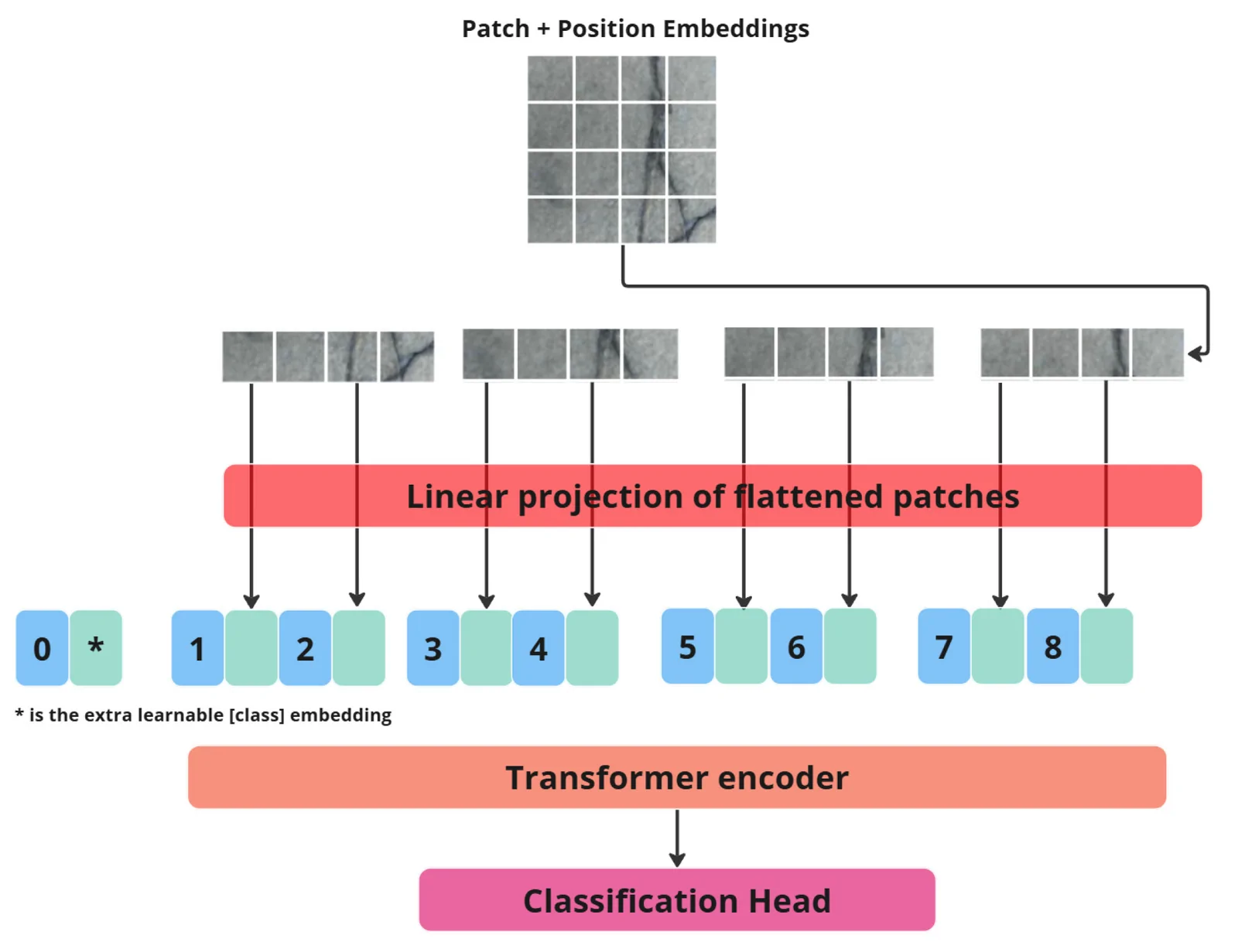
The vision transformer backbone.

**Figure 3:**
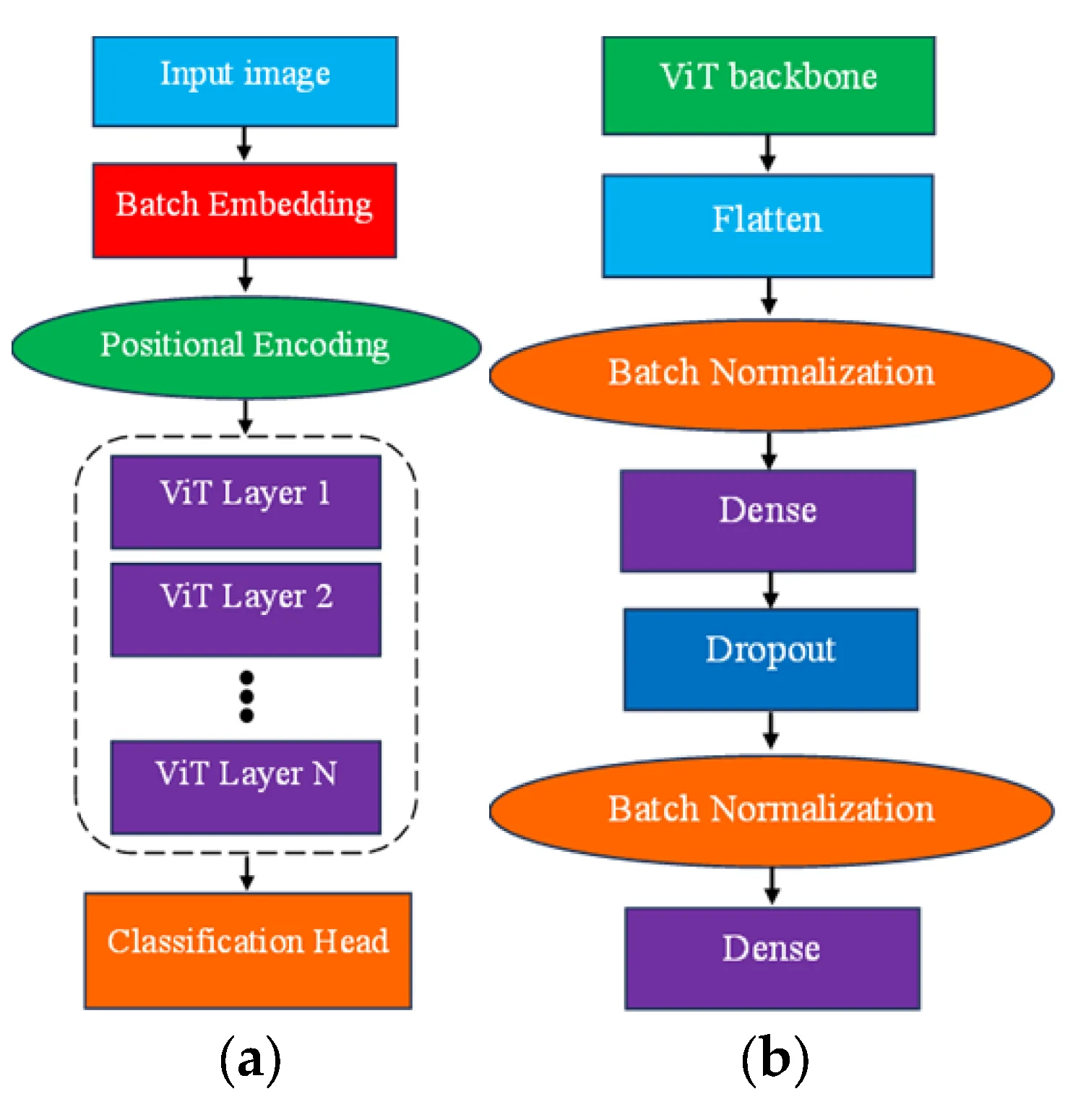
The vision transformer architecture: (**a**) the feature extraction head and (**b**) the vision transformer backbone with classification head.

ViTs are advanced learning technologies that can ingest images and perform complex classification tasks [55]. They can be thought of as partitioning images into small squares, and ViT then transforms these squares into a special code it can analyze [56]. In other words, the input image I(x,y) is segmented into S×S patches and then subsequently flattened, as in Figure 2 and Figure 3a. These flattened patches undergo linear embedding, transforming them into a sequence of tokens [57]. The model integrates positional embeddings with the token embeddings to incorporate spatial context. These positionally encoded patches are fed into a ViT encoder [58,59,60]. This encoder comprises three core components as:

1. The multi-head attention layers extract image features by focusing on specific image regions through an attention mechanism that aims to enhance the learning process.
2. The Add&Norm modules incorporate residual connections to alleviate vanishing gradients and include normalization to stabilize training.
3. Multilayer Perceptron (MLP) serves as the classification head within the ViT model.

The original classification head is replaced with the specialized head shown schematically in Figure 3b. The implemented head comprises flattening, batch normalization, a dense layer with 11 neurons and GELU activation, a dropout layer with a rate of 0.5 for ViT1 and ViT2, a second batch normalization layer, and a dense layer producing six class logits. ViT3 omits dropout and dense-layer L2 regularization. Softmax converts the logits to class probabilities. The 11-unit layer provides a compact intermediate mapping from the 768-dimensional ViT-B/32 representation to the six-class output. This width is a fixed design choice used in the experiments; no hidden-width ablation was performed, so it is not presented as an experimentally established optimum. Supervised training uses concrete surface images paired with their class labels.

### 3.3. Proposed Approach

**Figure 4:**
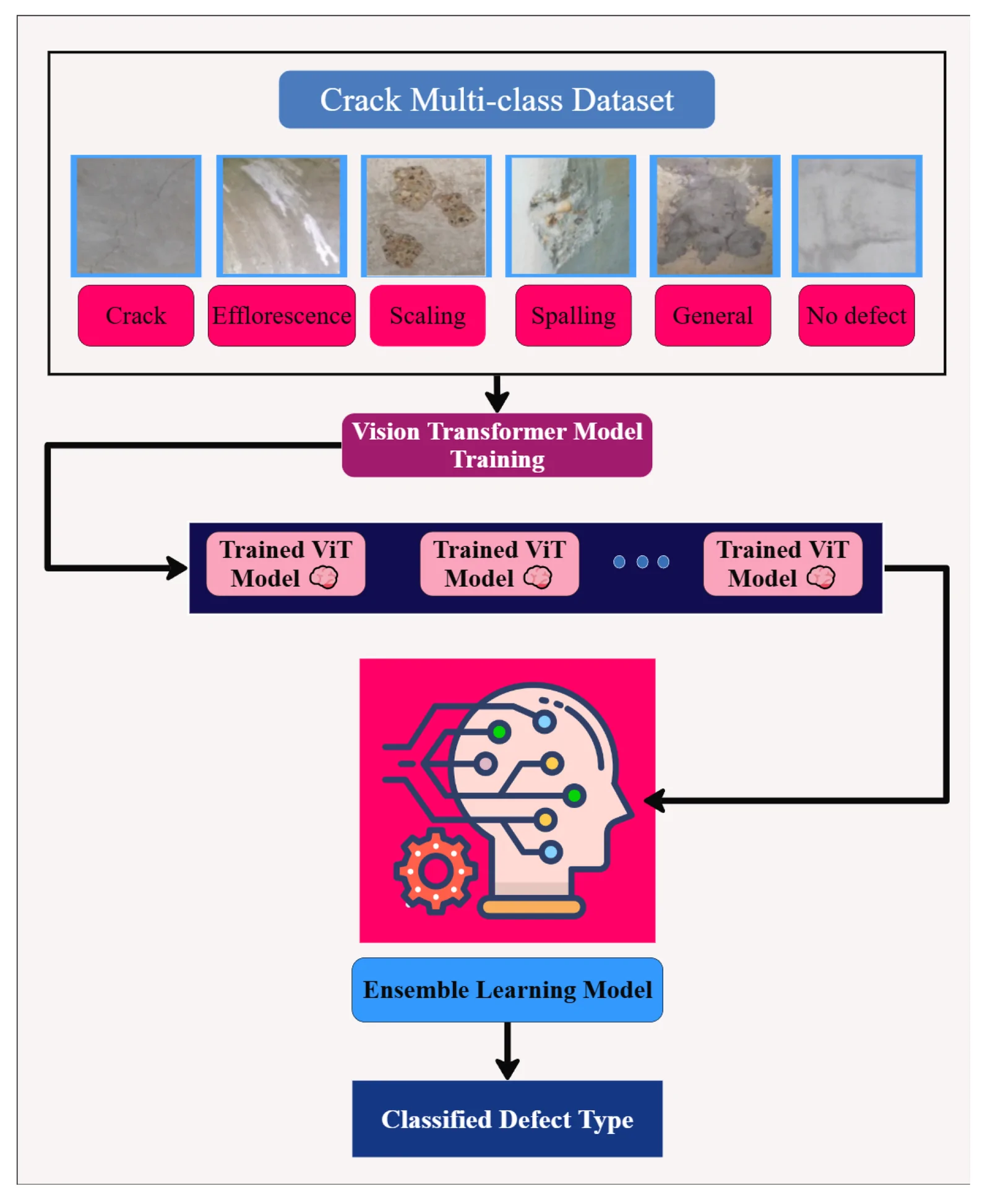
The proposed Co-ViTDefect approach.

As explained in previous sections, assessing cracks in concrete structures is essential in transportation and infrastructure engineering. Concrete structures are vulnerable to various defects throughout their service life, including spalling, cracks, scaling, efflorescence, and general deterioration. Effective identification and classification of these defects are essential to ensure safety. In this regard, a novel method that leverages ensemble ViT models is proposed. The technique called Co-ViTDefect helps to combine the predictions of multiple ViTs to achieve accurate multiclass defect detection, as shown in Figure 4. Co-ViTDefect starts by building a defect multiclass dataset of concrete structures, a diverse collection of images carefully labeled and categorized into distinct classes, such as spalling, cracks, scaling, efflorescence, general deterioration, and no defect. This well-established, carefully annotated dataset supports developing a highly precise classifier. The labeled images from the multiclass dataset are then used to train individual ViTs. The ViTs are trained to recognize different defect types. The ViTs can be trained on the same dataset, but their predictions will vary due to data shuffling, random initialization, and other factors. By leveraging multiple ViT models, the ensemble Co-ViTDefect model can mitigate individual-model biases and improve overall precision in multiclass defect detection.

The proposed Co-ViTDefect introduces Dirichlet ensemble learning (DEL) [61,62] for multiclass defect identification in concrete structures. DEL models the combination weights of core models as random variables drawn from a Dirichlet distribution. This Bayesian approach lets the ensemble capture uncertainty in model reliabilities and adaptively adjusts weights based on data evidence, while ensuring interpretability and probabilistic consistency. Unlike most traditional weighted ensemble methods, where weights among core models are either fixed or learned deterministically, DEL is Bayesian in spirit and models the combination weights as random variables following a Dirichlet distribution. The Dirichlet distribution is statistically valid and practically useful. Defined on the probability simplex, the Dirichlet enforces the simplex constraints: all weights are non-negative and sum to one. This ensures that the ensemble prediction remains a correct, probabilistically interpretable convex combination of base model outputs. However, a Dirichlet prior is not conjugate to the marginalized probability-mixture likelihood used in DEL; the posterior over the weights is therefore sampled numerically rather than obtained through a closed-form Dirichlet update. More importantly, the Dirichlet distribution conveniently represents and estimates epistemic uncertainty in ensemble weighting. Rather than settling on a single point estimate of model weights, DEL estimates a posterior distribution over model weights, representing the model’s belief regarding the faithfulness of each base learner.

Considering there are N core classifiers $C=[C_{1},C_{2},\ldots ,\;C_{N}]$, each classifier provides a predictive distribution $p\left(y|x,C_{N}\right)$, where ensemble learning aims to combine these predictions to achieve superior accuracy and robustness. The Dirichlet prior over the model weights can be defined as follows:

(1) $$w=\left[w_{1}\;,w_{2}\;,\ldots ,w_{N}\;\right]∼Dirichlet\left(\alpha \right),$$

where $\alpha =[\alpha_{1},\alpha_{2},\ldots ,\alpha_{N}]$ are concentration parameters encoding prior beliefs about model reliabilities. In our implementation, N = 3 (three ViT members are combined), and the prior is Dirichlet(1,1,1), which is uniform over the weight simplex. The Dirichlet prior naturally enforces the constraints $w_{i}\geq 0$ and $\sum_{i=1}^{N}w_{i}=1$, making it an ideal choice for the ensemble model. The ensemble predictive distribution is then calculated as:

(2) $$p\left(y|x,w\right)=\sum_{i=1}^{N}w_{i}p\left(y|x,C_{i}\right).$$

This formulation represents the ensemble as a convex combination of experts, with the Dirichlet distribution over the weights modeling epistemic uncertainty. In a Bayesian framework, considering that the dataset is represented as $D={\left\{\left[x_{j},y_{j}\right]\right\}}_{j=1}^{K}$, the posterior distribution over the weights given the observed data is required as follows:

(3) $$p\left(w|D\right)=\frac{p\left(D|w\right)\cdot p(w)}{p\left(D\right)},$$

where p(w) is the prior distribution over weights, p(D|w ) is the likelihood of the data given the weights, and p(D) is the marginal likelihood. The likelihood of observing this data under ensemble weights *w* can be calculated as:

(4) $$p\left(D|W\right)=\prod_{j=1}^{K}\sum_{i=1}^{N}w_{i}\cdot p\left(y_{j}|x_{j},C_{i}\right),$$

where for each data point $\left(x_{j},y_{j}\right)$, the ensemble predicts by mixing base model predictions, and the product over all data points represents the joint likelihood of the entire dataset under these weights. In DEL, D is the calibration subset, and the member probabilities are held fixed while the weights are inferred. Hence, the weights can be updated as in Equation (3). The posterior updating of weights using Bayesian updating over *w* combines the evidence from the data (via the likelihood) with the prior belief (Dirichlet prior), which can be represented as follows:

(5) $$p\left(w|D\right)\propto \left[\prod_{j=1}^{K}\sum_{i=1}^{N}w_{i}\cdot p\left(y_{j}|x_{j},C_{i}\right)\right]\cdot Dirichlet\left(w|\alpha \right),$$

where the likelihood term $\prod_{j=1}^{K}\sum_{i=1}^{N}w_{i}\cdot p\left(y_{j}|x_{j},C_{i}\right)$ pulls the weights towards models that better explain the observed data, the Dirichlet prior Dirichlet(w|α) acts as a regularizer that prevents overconfidence in any single model unless strongly supported by data, and the resulting posterior distribution represents an updated belief over how much trust can be given to each core model after observing data.

Since the mixture likelihood in Equation (4) is a product of weighted sums, Equation (5) does not reduce to a Dirichlet distribution. Because of this, a simple Dirichlet posterior update is not used. Posterior inference uses an additive log-ratio transformation with N − 1 (in our case two) unconstrained coordinates, and the Jacobian is included explicitly (Equation (6a)).

(6a) $$z_{i}=log\left(\frac{w_{i}}{w_{N}}\right),\;i=1,\ldots ,N-1,\;\;\;w=softmax\left(z_{1},\ldots ,z_{N-1},0\right),\;\;\;\left|J\right|=\prod_{i=1}^{N}w_{i},$$

which gives the log target density sampled in the unconstrained coordinates (Equation (6b)).

(6b) $$log\pi_{z}\left(z\right)=\sum_{j=1}^{K}log\left[\sum_{i=1}^{N}w_{i}\;p\left(y_{j}|x_{j},C_{i}\right)\right]+\sum_{i=1}^{N}\alpha_{i}logw_{i}+const,$$

The coefficient of log w_i_ in the transformed target is α_i_ because the prior contributes α_i_ − 1 and the Jacobian contributes one. Four random-walk Metropolis chains are run with 3000 warm-up iterations and 4000 retained draws per chain. Proposal adaptation occurs only during warm-up. The retained draws are transformed back to simplex weights, and the posterior mean is used for probability fusion:

(6c) $$\bar{p}\left(y|x\right)=E_{w|D}\left[\sum_{i=1}^{N}w_{i}\;p\left(y|x,C_{i}\right)\right]=\sum_{i=1}^{N}E\left[w_{i}|D\right]p\left(y|x,C_{i}\right).$$

This equality is exact for the linear mixture, so prediction does not require running MCMC for each new image.

### 3.4. Dataset

In this study, a multiclass concrete structures defect dataset [63] is utilized to develop the proposed Co-ViTDefect approach. The image dataset includes images of concrete structure surfaces with various defect types. These images are categorized into six distinct classes: cracks, efflorescence, general, no defect, scaling, and spalling. The dataset comprises 2411 images distributed across the following classes: 789 images of cracks, 311 images of efflorescence, 264 general defect images, 452 images with no defects, 168 images of scaling, and 427 images of spalling. For the training and evaluation of the proposed Co-ViTDefect, a preprocessing step to check conflicting label files and extra duplicates is applied. As a result, only 2396 images pass; 10 are excluded due to conflicting labels, and 5 are discarded because they appear in more than one class folder. Table 1 describes the dataset details.

In all experiments, we use 2396 images under 5-fold group cross-validation. Within each folder, 20% is held out as a test set, while the rest (80%) is kept for (~60%) training and (~10%) early stop validation, and a separate calibration subset (~10%) is used to fit the ensemble weights and temperatures. Table 2 gives the exact number of images per split for fold 1.

After that, two standard image-appropriate mechanisms, online augmentation and class-weighted cross-entropy, are utilized. Online augmentation is applied to training images: Each ViT member uses its own random flips, rotations (10–15°), and zoom (±10–20%), generated freshly at each epoch. Validation, calibration, and test images are never augmented. We apply class-weighted cross-entropy separately to each fold’s training subsets, weighting each class by N/(k × nk), where nk is the number of training samples per class; thus, minority classes such as the “scaling” class carry more weight than the “crack” class.

**Figure 5:**
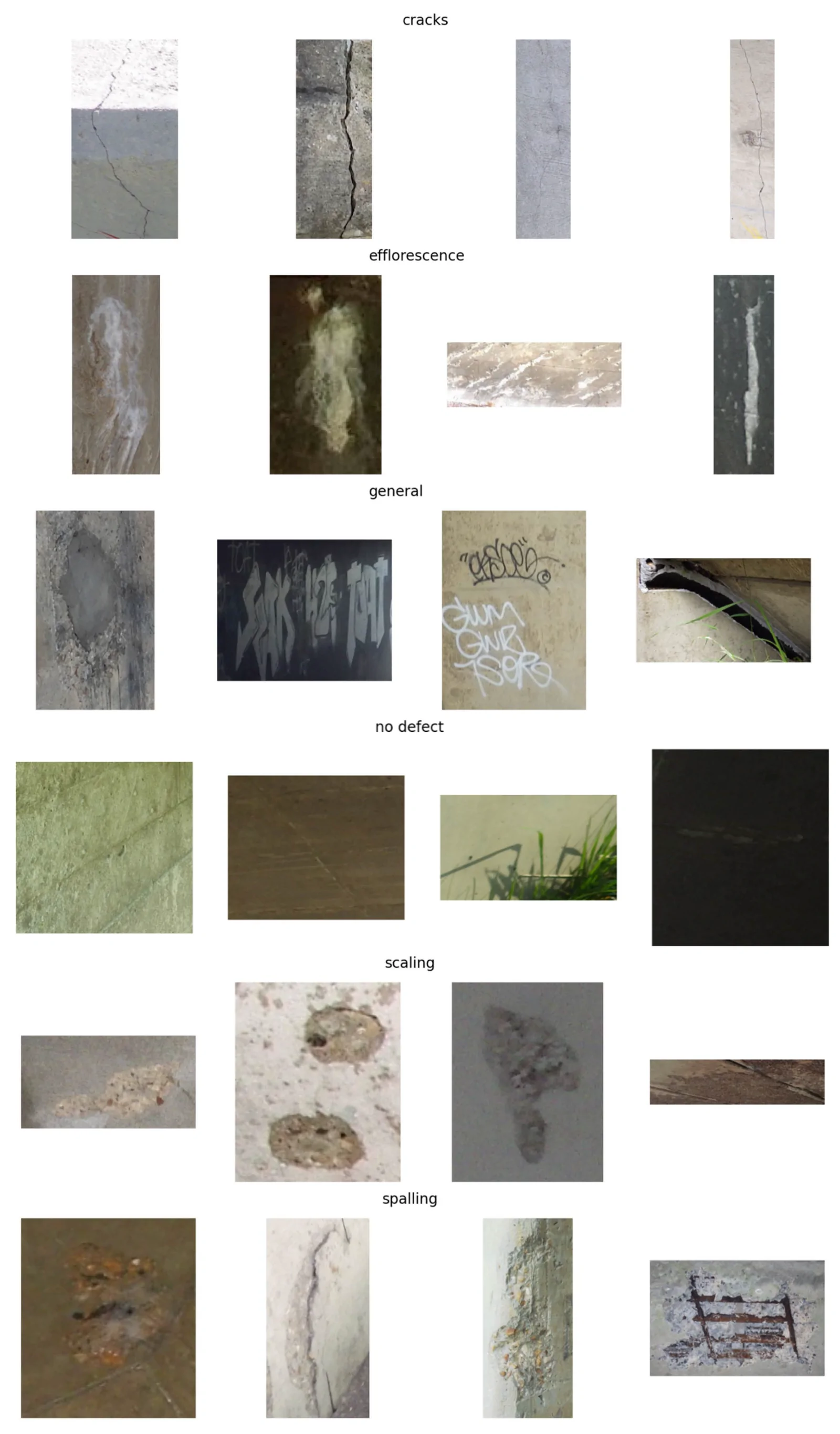
Visual examples from the utilized dataset.

In addition, the training parameters are set as follows: Input size is set to 224 × 224 × 3, three training seeds, batch size is set to 256, training epochs are set to 50, learning rate (lr) is set to 1 × 10^−4^, optimizer is set to Adam Optimizer, label smoothing (to prevent overfitting) is set to 0.2, training monitor is set to validation loss, reduce learning rate factor is set to 0.2, reduce learning rate patience is set to 3, reduce learning rate (min_delta) is set to 1 × 10^−4^, reduce learning rate (min_lr) is set to 1 × 10^−6^, and early stop condition patience is set to 10. Some visual examples of the dataset are given in Figure 5.

### 3.5. Development of the Co-ViTDefect Model

We train three ViT models to develop the Co-ViTDefect concrete-structure defect metaclassifier. Although the individual models share the same ViT-B/32 backbone and classification head design, the diversity is introduced through different random seeds, augmentation settings, and regularization. ViT1 and ViT2 use dropout of 0.5 and L2 regularization of 0.01, while ViT3 drops these steps. In this case, the methodology is a homogeneous ViT ensemble. The multi-classifier Co-ViTDefect ensemble leverages ViT’s representational power, using three distinct configurations based on the base ViT architecture. Such an architecture typically processes input images by dividing them into fixed-size patches, linearly embedding them, and feeding them as sequences, similar to the approach used in natural language processing. These patch embeddings are passed through a sequence of Vit encoder layers, which enables the model to capture the global dependencies and patterns of the image through self-attention mechanisms. In ViT_model1, the base vit-b32 Transformer encoder produces a 768-dimensional feature vector. The output is then flattened, passed through batch normalization, and then through a dense layer with 11 output units. A dropout layer is applied for regularization, followed by batch normalization and an output dense layer with six units to map the dataset’s six defect categories. This ViT_model1 setup has an exceptionally high parameter count of 87,466,879, indicating high capacity to learn detailed features of concrete structure surface images. All three members use Adam with an initial learning rate of 1 × 10^−4^, a maximum of 50 epochs, and early stopping after 10 epochs without an improvement in validation loss. ReduceLROnPlateau multiplies the learning rate by 0.2 after three epochs without improvement, with min_delta = 1 × 10^−4^ and a minimum learning rate of 1 × 10^−6^. Member diversity comes from independent training seeds, member-specific augmentation, and the regularization differences described above. The same 11-unit GELU hidden layer and 6-unit output are retained in all three members.

Multiclass classification models are evaluated using True Positives (TP), False Positives (FP), True Negatives (TN), and False Negatives (FN) [64]. Precision, recall, F1-score, AUC, and accuracy are used to evaluate the core and Co-ViTDefect models. Precision is the ratio of TPs to the sum of TPs and FPs, as shown in Equation (7). In contrast, recall considers FN errors and is calculated as the ratio of TPs to the sum of TPs and FNs, as in Equation (8). Equation (9) calculates the F1-score, which measures both recall and precision. Additionally, the area under the ROC curve (AUC) assesses the model’s ability to distinguish between positive and negative predictions across all possible classification thresholds. It can be found by plotting the True Positive Rate (TPR) against the False Positive Rate (FPR) at various thresholds. Then, the area under the resulting curve can be calculated as the AUC. Accuracy is computed as Equation (10) illustrates, where CM is the six-class confusion matrix, and CM_ij_ is the number of test images whose true class is “I” and whose predicted class is “j” (where i,j = 1,…,6). Macro precision, macro recall, and macro F1 are unweighted averages of the corresponding class-wise quantities. Macro F1 (Equation (11)) is the average of class F1 scores and is not generally the harmonic mean of macro precision and macro recall. In this single-label multiclass task, support-weighted recall equals overall accuracy. All metrics are computed by an automated script from saved test probabilities and labels, rather than from manually entered values. It checks that probabilities are finite and sum to one, that confusion matrix counts equal the test set size, that diagonal counts reproduce accuracy, and that weighted recall equals accuracy. The saved predictions contain exactly one out-of-fold entry per retained image for each of the 15 raw or calibrated methods, with no duplicate image entry within a method.

(7) $$Precision_{k}=\frac{TP_{k}}{TP_{k}+FP_{k}}$$

(8) $$Recall_{k}=\frac{TP_{k}}{TP_{k}+FN_{k}}$$

(9) $$F1_{k}=\frac{2\cdot Precision_{k}\cdot Recall_{k}}{Precision_{k}+Recall_{k}}$$

(10) $$Accuracy=\frac{\sum_{k=1}^{6}{CM}_{kk}}{\sum_{k=1}^{6}\sum_{j=1}^{6}{CM}_{ij}}$$

(11) $$Macro\;F1=\frac{1}{6}\sum_{k=1}^{6}{F1}_{k}$$

## 4. Results and Discussion

In this section, the total experimental outcomes of the multiclass defect classification of concrete structures are presented under two themes: individual models and the developed Co-ViTCrack approach. Results are presented for the three individual ViT models and the proposed Co-ViTDefect ensemble. The training process is performed in five folds with a training split of 60%, 10% for validation, 10% for calibration, and 20% for test. In this order, the exact counts were 1437/240/240/479 in folds 0, 3, and 4; 1437/239/240/480 in fold 1; and 1438/240/239/479 in fold 2. We split the data before augmentation and applied it only to the training images. The main experiment combined conventional image augmentation with class-weighted training, while we later used SOMTE only for comparison. Results are reported as the mean and sample standard deviation (SD) across five test folds. Class-level tables, confusion matrixes, and ROC curves use pooled out-of-fold predictions, with one held-out prediction for each image. The pooled values are reported separately from fold averages.

### 4.1. Individual Models

**Figure 6:**
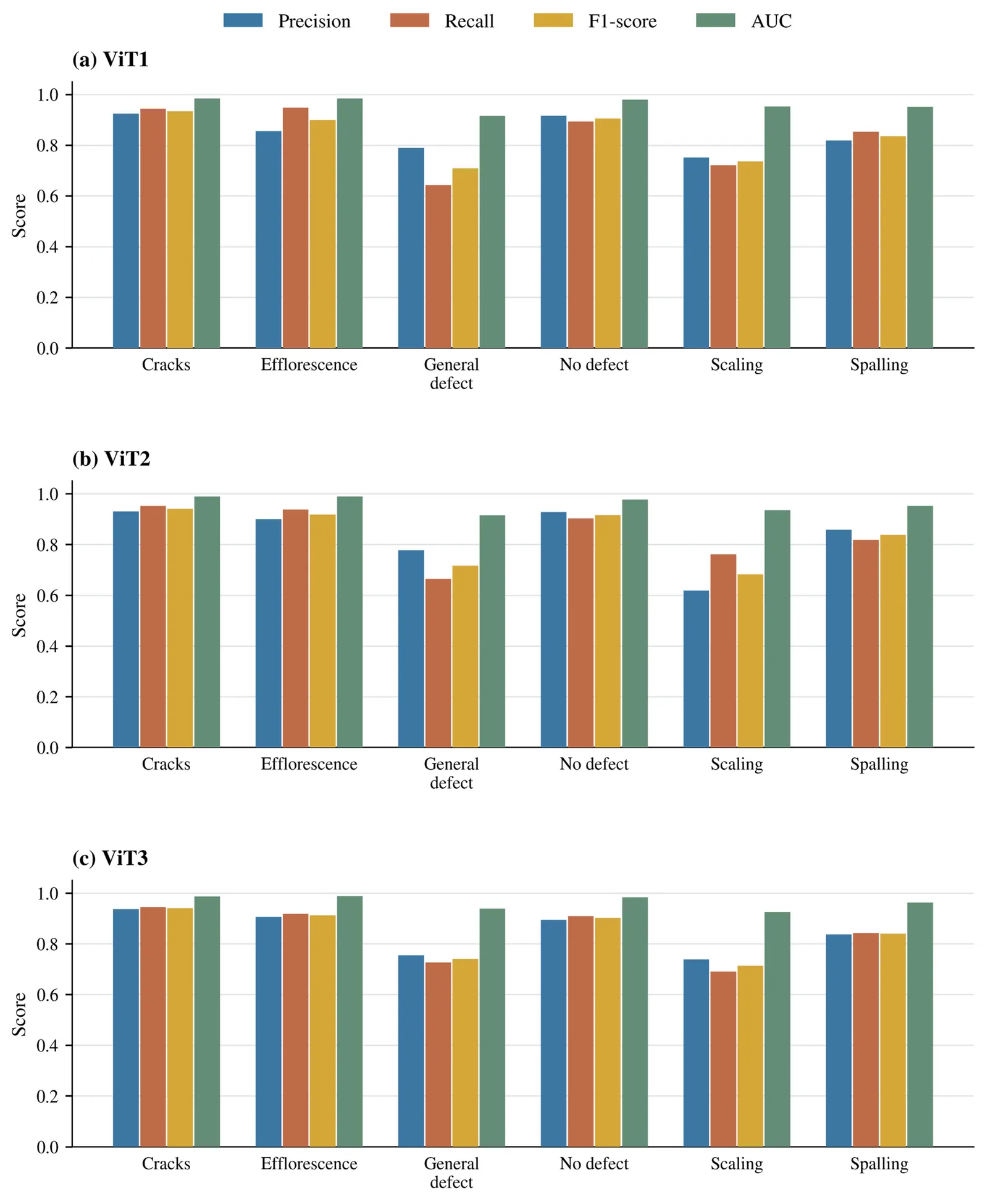
Class-level precision, recall, F1-score, and one-versus-rest AUC for the three ViT members, calculated from pooled out-of-fold test predictions.

**Figure 7:**
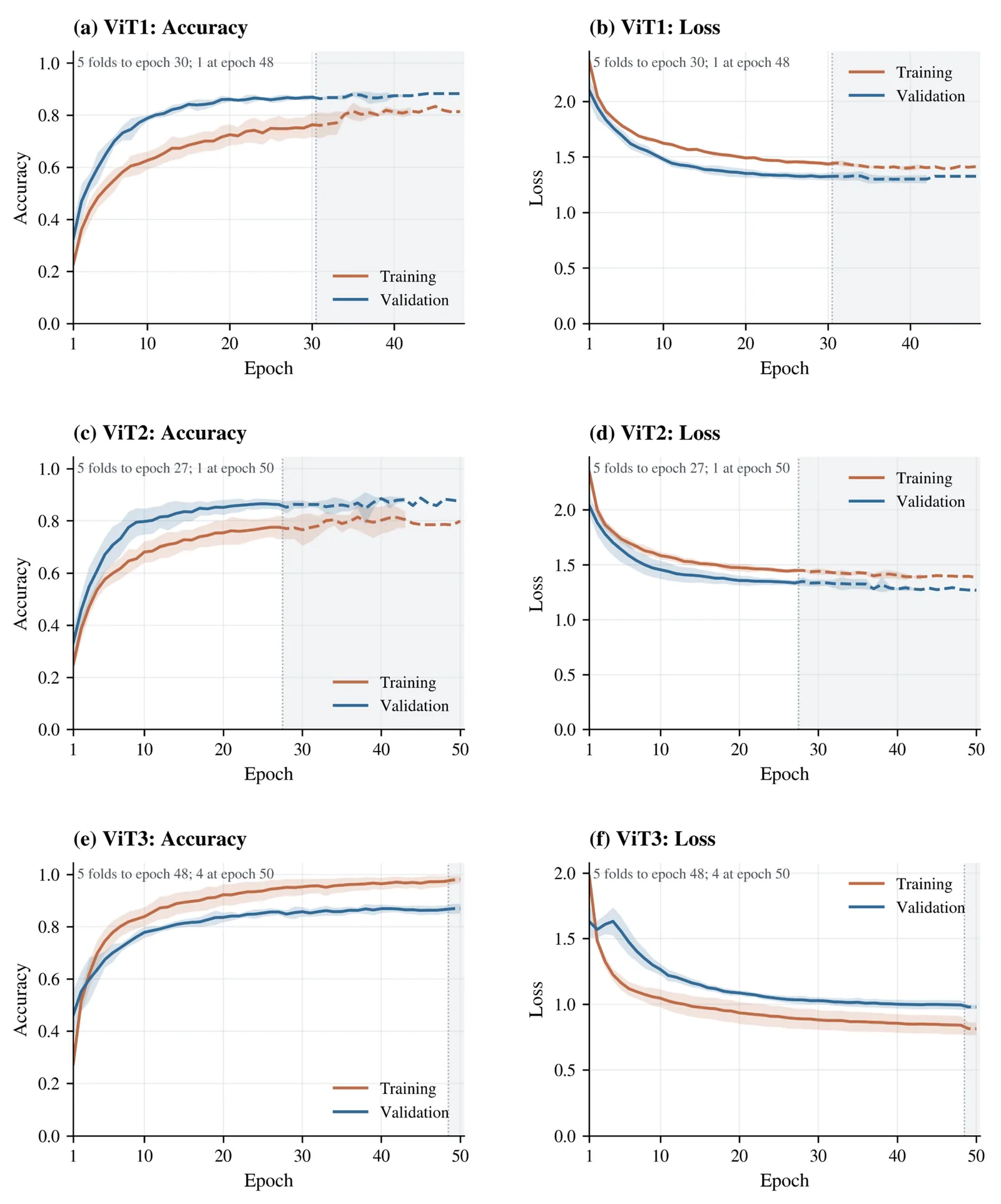
Train and validation accuracy and loss across five folds for all individual models.

After training the three individual models that serve as the core of the Co-ViTDefect model, the three trained ViT models are evaluated. ViT1, ViT2, and ViT3 achieved mean test accuracies of 86.98%, 87.19%, and 87.48%, with SD values of 2.31, 1.56, and 0.89 percentage points, respectively. The corresponding macro F1 scores were 83.62%, 83.51%, and 84.18%, respectively. The ViT3 model produced the highest mean accuracy with the smallest variation in accuracy among all individual models. Figure 6 and Table 3 show the class-level results, where the general defect and scaling classes were the most difficult to predict compared with the cracks and no-defect categories. Figure 7 shows the mean training and validation curves across five folds, with shaded bands indicating the SD values across different folds. Since some training processes ended before the maximum 50 epochs (due to early stop condition), the averaging operation of training and validation values was computed from the remaining recorded training histories. Lines in all curves represent the average values, while the shaded bands show one sample standard deviation. Solid lines include all five folds, and dashed segments include only folds still training. When only one fold remains, shading is omitted from the curve. No values were extended beyond the recorded stopping epoch. Validation data were used for model selection; the test data were not used to select an epoch. Training loss includes the effects of class weighting, label smoothing, and regularization. Training and validation curves of the individual models shown in Figure 7 prove that each model’s convergence is different from others, so a fusion (ensemble) of them can significantly improve performance.

**Figure 8:**
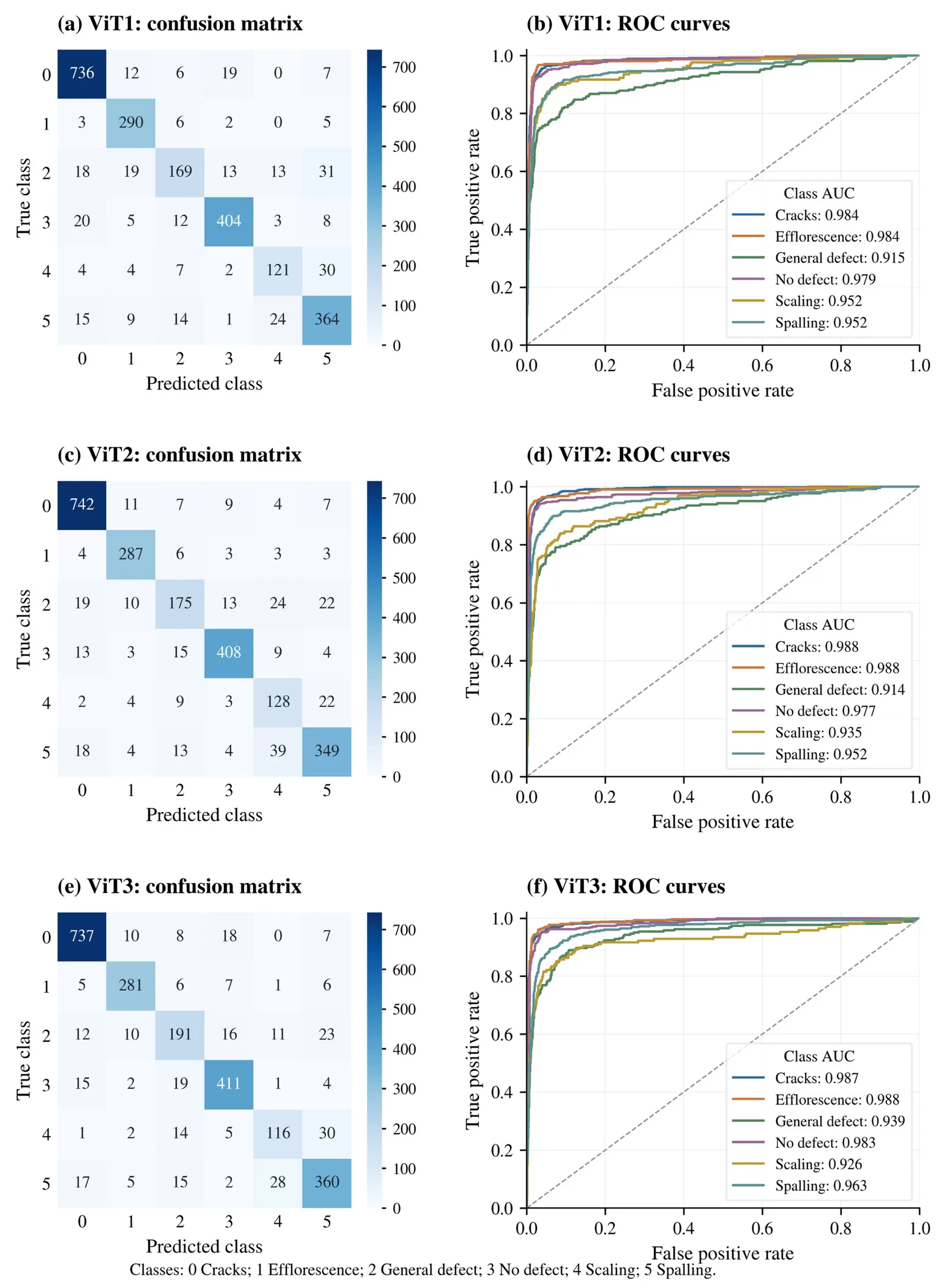
The confusion matrices and ROC curves for the three ViT models.

Figure 8 presents the pooled confusion matrices and one-versus-rest ROC curves. All results support the idea that the three individual ViT models perform differently. ViT2 recognizes “efflorescence” and “no defect” best (F1: 91.84% and 91.48%), while ViT3 recognizes “crack,” “general defects,” and “spalling” best (F1: 94.07%, 74.03%, and 84.01%). In contrast, ViT1 recognizes “scaling” best (73.56%).

Although the individual models share the same ViT-B/32 backbone, they differ in many aspects. Table 4 clarifies the pairwise test-error diversity across all folds and proves the real difference between them despite using the same training strategy and the same data. Table 4 shows that the proposed ViT members disagree on almost 10.6–11.2% of test predictions, and the error correlation registered an approximate range of 0.59–0.61. These results support the efficiency of the ViT ensemble.

Attention maps help explore the model’s ability to focus on the essential parts of the image. Table 5 compares the attention maps of the three ViT models. Although all trained models can focus on the crack or defect region in the input image, ViTmodel3 performs best. This finding is also supported by class activation maps (CAMs), which highlight the discriminative region of the concrete crack image and show the most essential parts of the input the model uses to make its decision. In the first example, the CAMs effectively highlighted the crack regions, especially for ViTmodel3. For the second example (spalling type), the second and third models (ViTmodel2 and ViTmodel3) marked the two spalling parts. In contrast, in the third example, ViTmodel3 effectively highlighted the scaling and crack regions. ViTmodel2 and ViTmodel3 succeeded in marking the distributed spalling areas, with a slight edge to ViTmodel2 (worked better in the case of distributed defect regions). CAMs and attention map plots of the trained models show their ability to focus on concrete defects in the input images.

### 4.2. Co-ViTDefect Model’s Results

**Figure 9:**
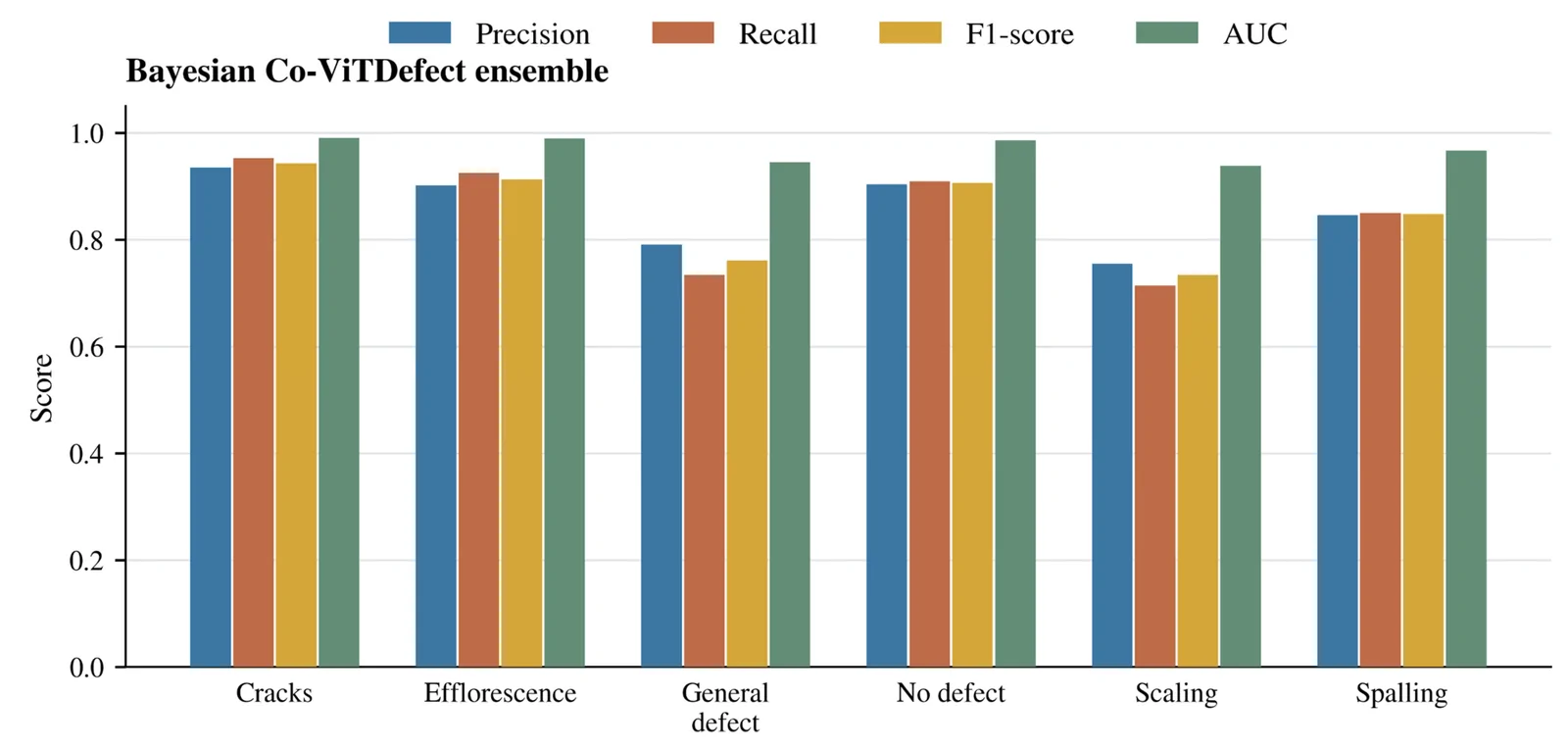
Statistical metrics of the Co-ViTDefect model.

**Figure 10:**
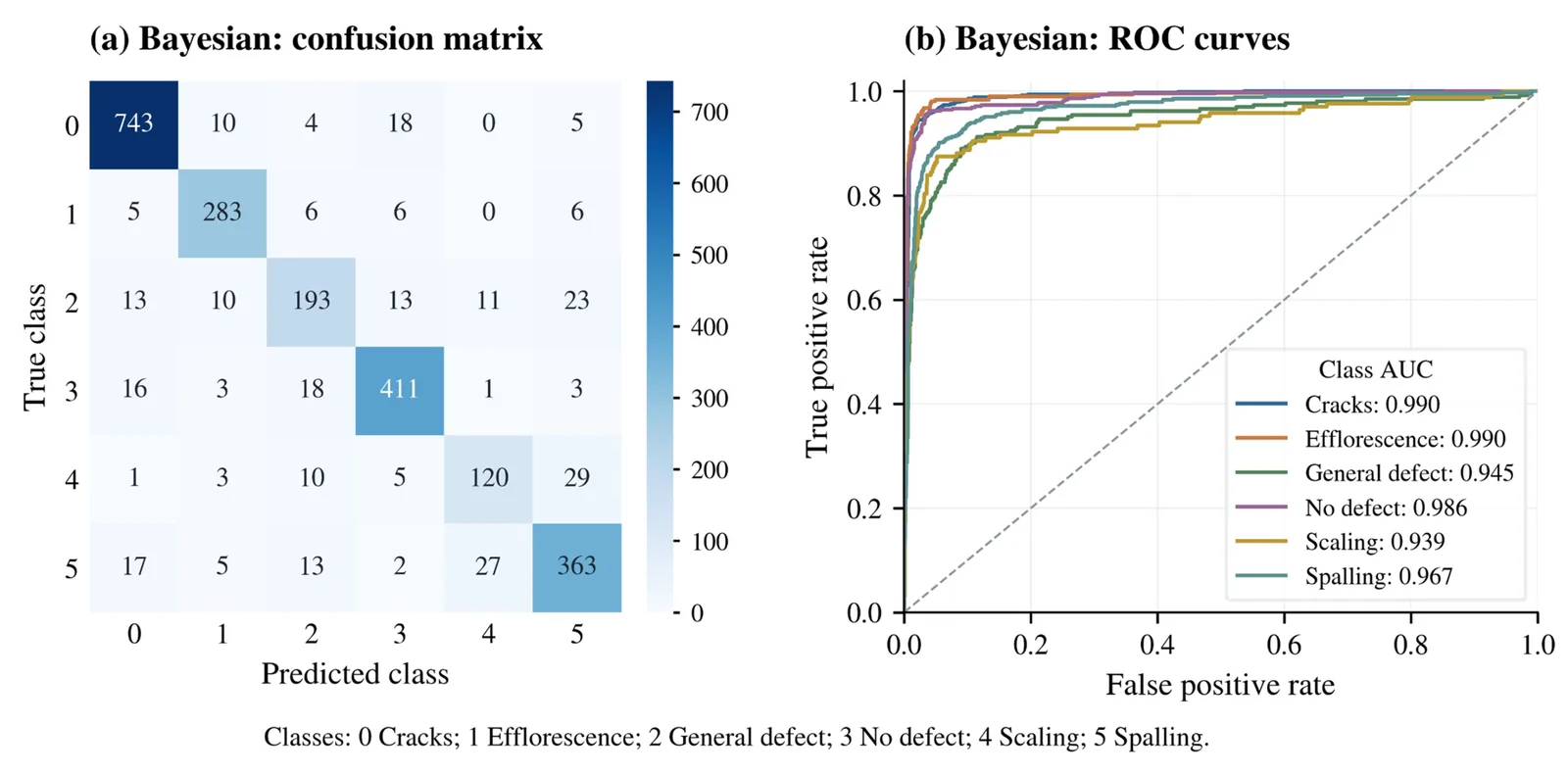
Pooled out-of-fold results for the Bayesian Co-ViTDefect ensemble: (**a**) confusion matrix and (**b**) ROC curve.

In order to test the performance of the proposed Co-ViTDefect, a weighted ensemble with Bayesian mixture weights is implemented. A uniform Dirichlet (1, 1, 1) prior was assigned to the weights [65], while weights are estimated using calibration data only. This prior is not conjugate to the stated mixture likelihood. The posterior was therefore estimated using four random-walk Metropolis chains in additive log-ratio coordinates, including the transformation Jacobian (3000 warm-up and 4000 retained draws each). The posterior mean weights were used to obtain the final mixture probabilities. Figure 9 and Figure 10 show the statistical metrics of the Co-ViTDefect ensemble pipeline for all six categories, and the corresponding confusion matrix and ROC plots. Metrics indicate that the main improvement of using the ensemble model is in the “general defect” class, in which the ensemble recognized 193 of 263 images (73.4%), while the three ViT models recognized 169 (64.3%), 175 (66.5%), and 191 (72.6%). The ensemble model also achieved a slight improvement in the “crack” category (743 of 780 samples with an accuracy of 95.3%) against the best individual model (736 of 780). Results in Table 6 and Table 7 prove that the Bayesian ensemble achieved a mean test accuracy of 88.19% with a standard deviation of 1.41% and a mean macro F1-score of 85.12% with a standard deviation of 1.8%, outperforming the three individual models by 1.21%, 1%, and 0.71%, respectively, indicating the difference between these three individual architectures (when a model misses, maybe another one succeeds). Moreover, the ensemble model showed less fold-to-fold variation than the individual ViT1 and ViT2 models.

### 4.3. Visual Comparison Between All the Models

**Figure 11:**
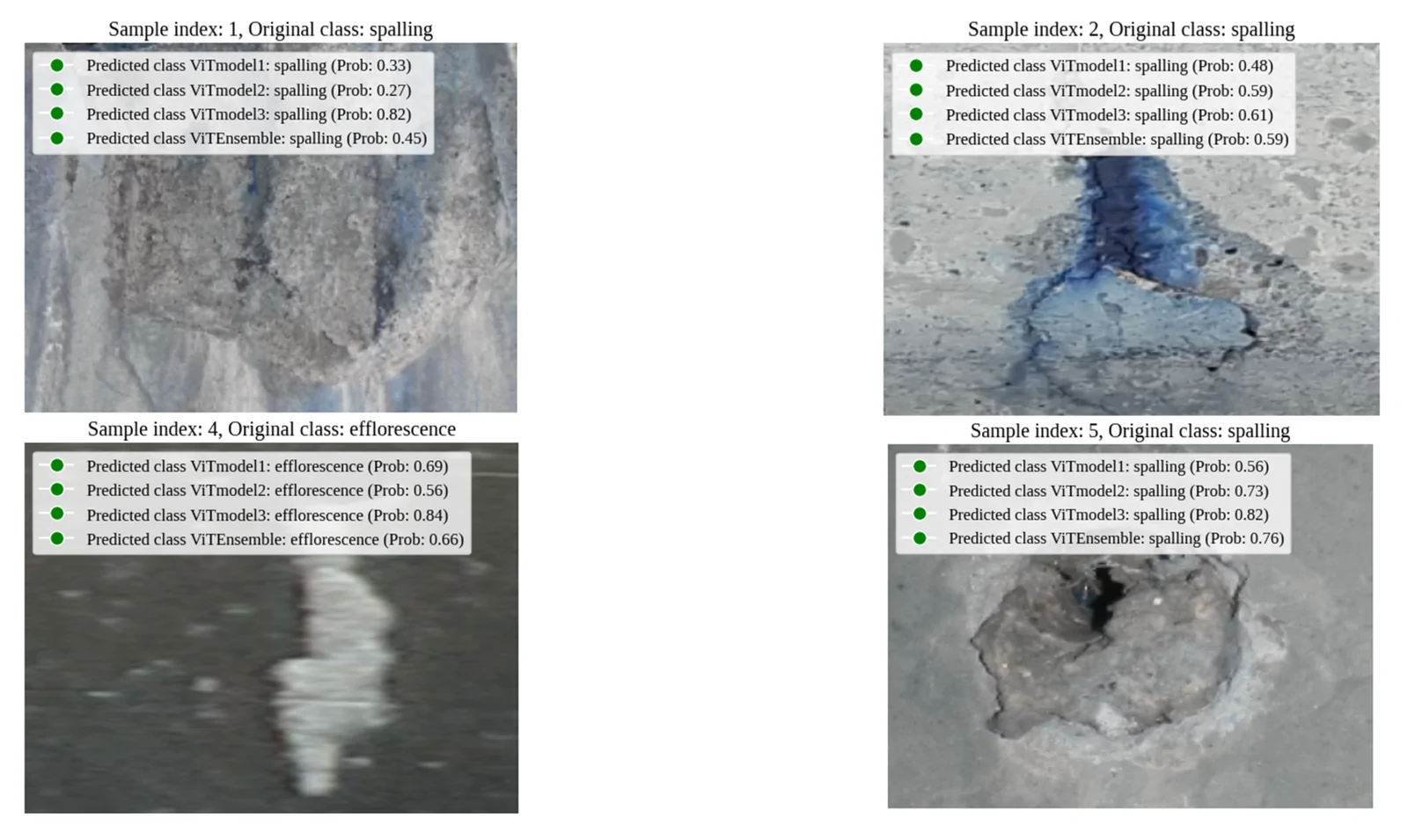

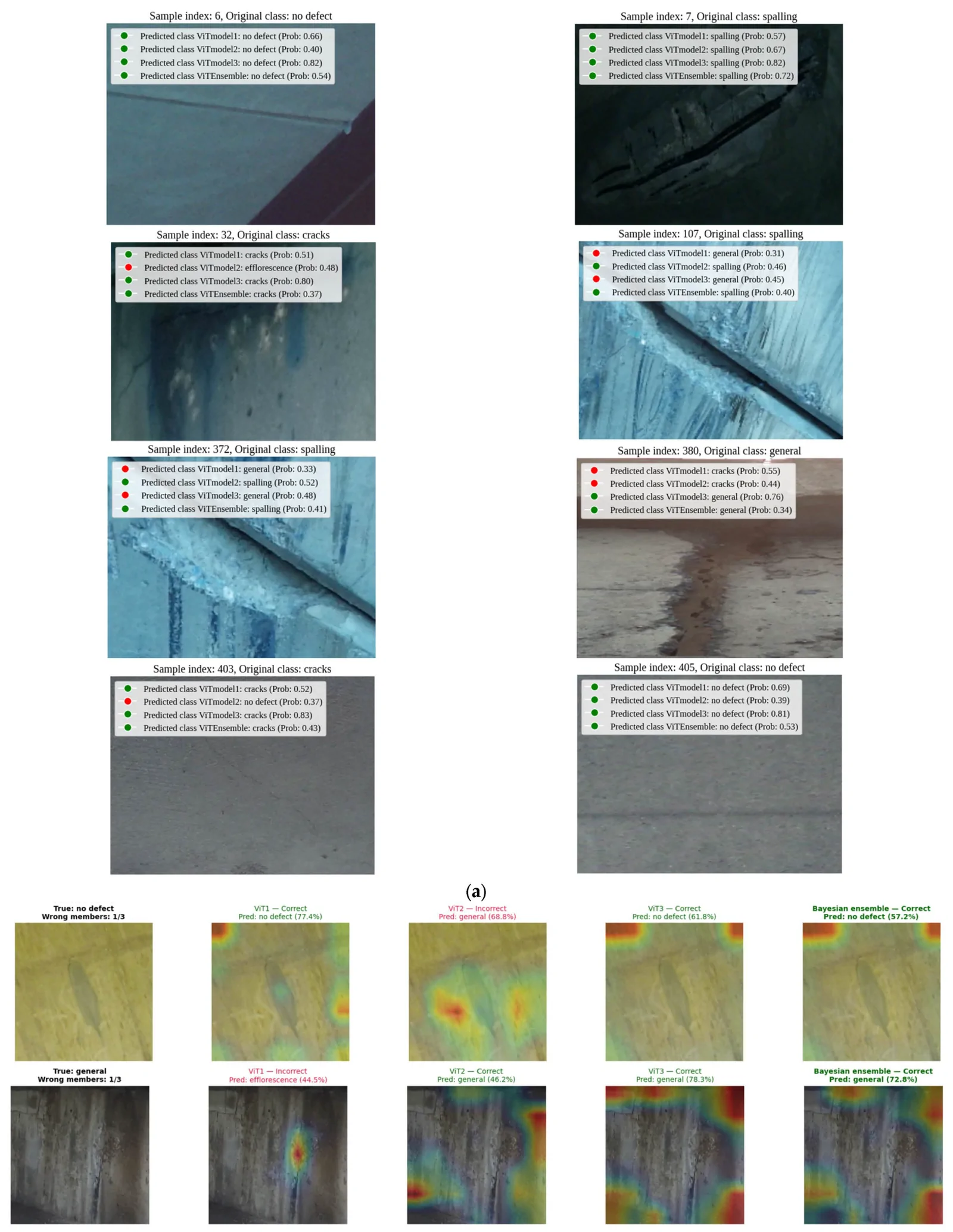

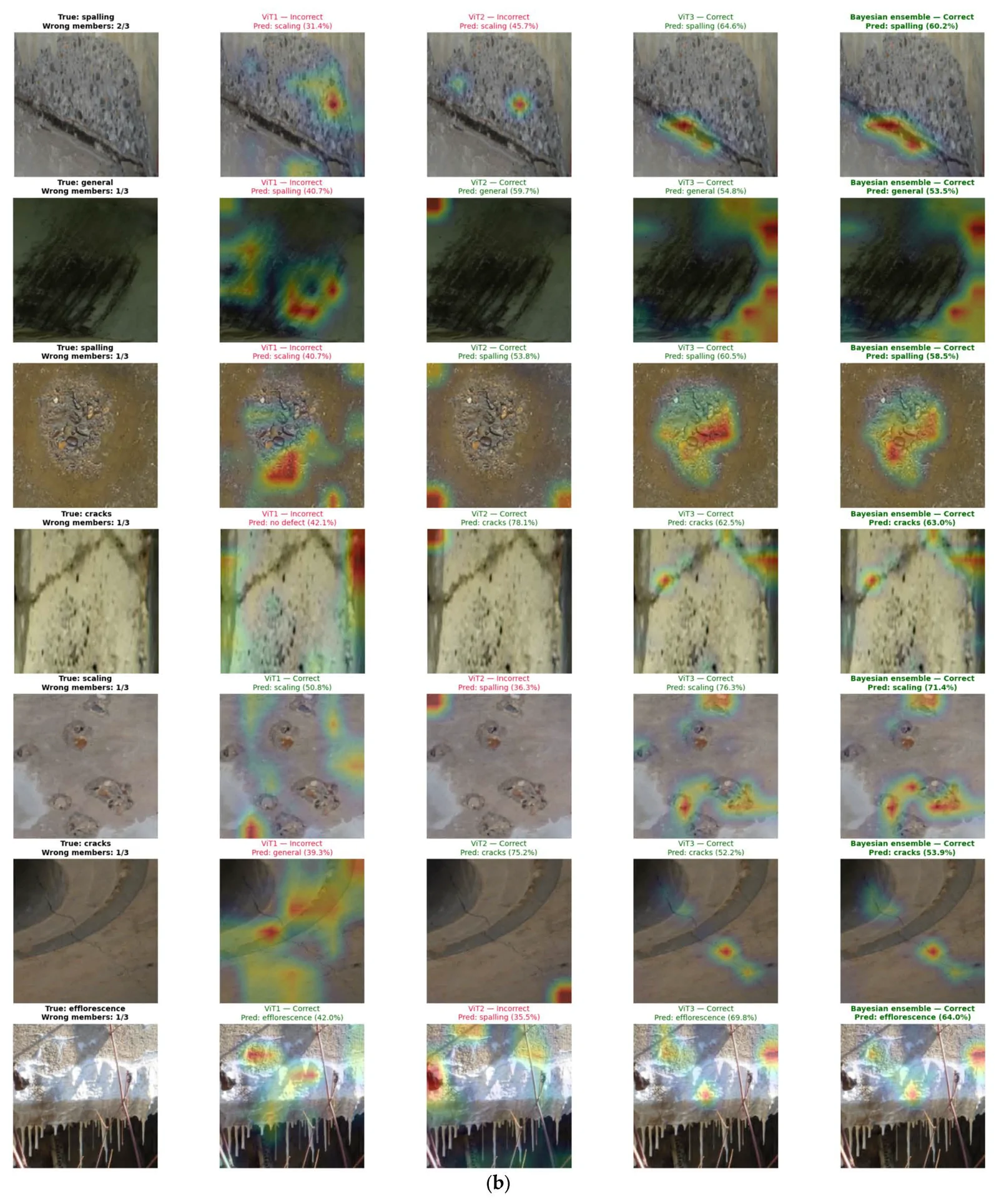
Visual results of Co-ViTDefect ensemble: (**a**) general comparison and (**b**) using Gra-CAM.

To draw a clear picture of the performance of the Co-ViTDefect with the other core models, a visual comparison using randomly chosen samples from the dataset is conducted as in Figure 11. Across the broader collection of samples, patterns observed in the original samples are again reinforced, showing the discrepancy between prediction confidence and accuracy among the individual ViT models and the benefit derived by the Co-ViTDefect approach. In the cases of spalling (Samples 1, 2, 5, 7, 107, 372), we consistently find variability in the confidence and precision of single models; however, the Co-ViTDefect adequately predicts “spalling” more frequently with more confidence than some individual models (e.g., Sample 2, 5, 7, 107) and, in cases where single models display lower confidence or disagreement (e.g., Sample 1), the Co-ViTDefect prediction reflects a more balanced result. In Sample 4 (original class: efflorescence), the Co-ViTDefect accurately predicts “Efflorescence,” demonstrating its ability to use the correct prediction from one of the stronger individual models (ViT_model3) even when others get it wrong. Sample 6 (original class: no defect) also demonstrates the ensemble’s ability to produce a correct prediction (“No defect”) even when individual models are incorrect with varying degrees of confidence. For Sample 32 (original class: cracks), all the models give a highly confident prediction, repeated and reaffirmed by the ensemble. ViT_model1 and ViT_model3 incorrectly predict “General” with medium confidence, while ViT_model2 predicts “Cracks.” Here, the Co-ViTDefect accurately predicts “General” with a medium probability, indicating its utility in bringing together heterogeneous model outputs for correct classification for this problematic category. This makes Sample 380 (original class: general) especially informative. Sample 403 (original class: cracks) is a case of correct predictions by each of the individual models, and the Co-ViTDefect rightly predicts “cracks” with confidence as well. Finally, Sample 405 (original class: no defect) has correct predictions by ViT_model1 and ViT_model3, whereas ViT_model2 wrongly predicts “cracks”; the Co-ViTDefect rightly predicts “No defect” with reasonable confidence, again showing its resilience to mistakes by individual models. Overall, the increased visual comparison across these diverse samples strongly favors the advantages of the Co-ViTDefect method. The Co-ViTDefect tends to show an ability to either supplement correct predictions by outstanding individual models, recover misclassifications by a group of models by leveraging others’ expertise, and make a more robust and, in many instances, more confident overall prediction, significantly improving performance on the challenging “General” class. These qualitative instances, coupled with the quantitative results, strongly suggest the strength of the Co-ViTDefect on this concrete defect classification task. Figure 11b, on the other hand, shows how the ensemble model effectively detects different concrete defect types and normal cases. Grad-CAM highlights image regions that contribute most to the model’s prediction. For most defect images, the highlighted regions fall on the defect itself. For the general and no-defect cases, the highlighted regions form a specific pattern.

### 4.4. Ablation Study: Calibration and Uncertainty

Table 8 shows that the proposed Co-ViTDefect achieved the third-best accuracy (88.19 ± 1.41) among ensemble models, including uniform averaging (89.57 ± 1.33) and majority voting (89.31 ± 1.39). However, this result does not prove that these two ensemble strategies are always the best, since the uniform averaging ensemble gives equal weight to all ViT individual models, which is not suitable for all cases. Majority voting ensemble returns only class labels without probabilities, so its calibration cannot be measured. Moreover, all members receive equal weight, reducing its reliability and trustworthiness.

Temperature scaling was fitted on the calibration subset and evaluated on the held-out test subset. Negative log-likelihood (NLL), Brier score, and expected calibration error (ECE) are all presented in Table 9, where lower values indicate better performance. ECE used 15 equal-width confidence bins, and the multiclass Brier score was summed across the six classes for each image. As shown in Table 9, temperature scaling reduced the Bayesian ensemble ECE from 0.214 to 0.042 and NLL from 0.570 to 0.403.

General defects and scaling remained the main class-level weaknesses. For the Bayesian ensemble, their mean recalls were 73.37% and 71.52%, respectively. Temperature scaling reduced the mean one-versus-rest ECE from 0.063 to 0.028 for general defects and from 0.068 to 0.028 for scaling.

**Figure 12:**
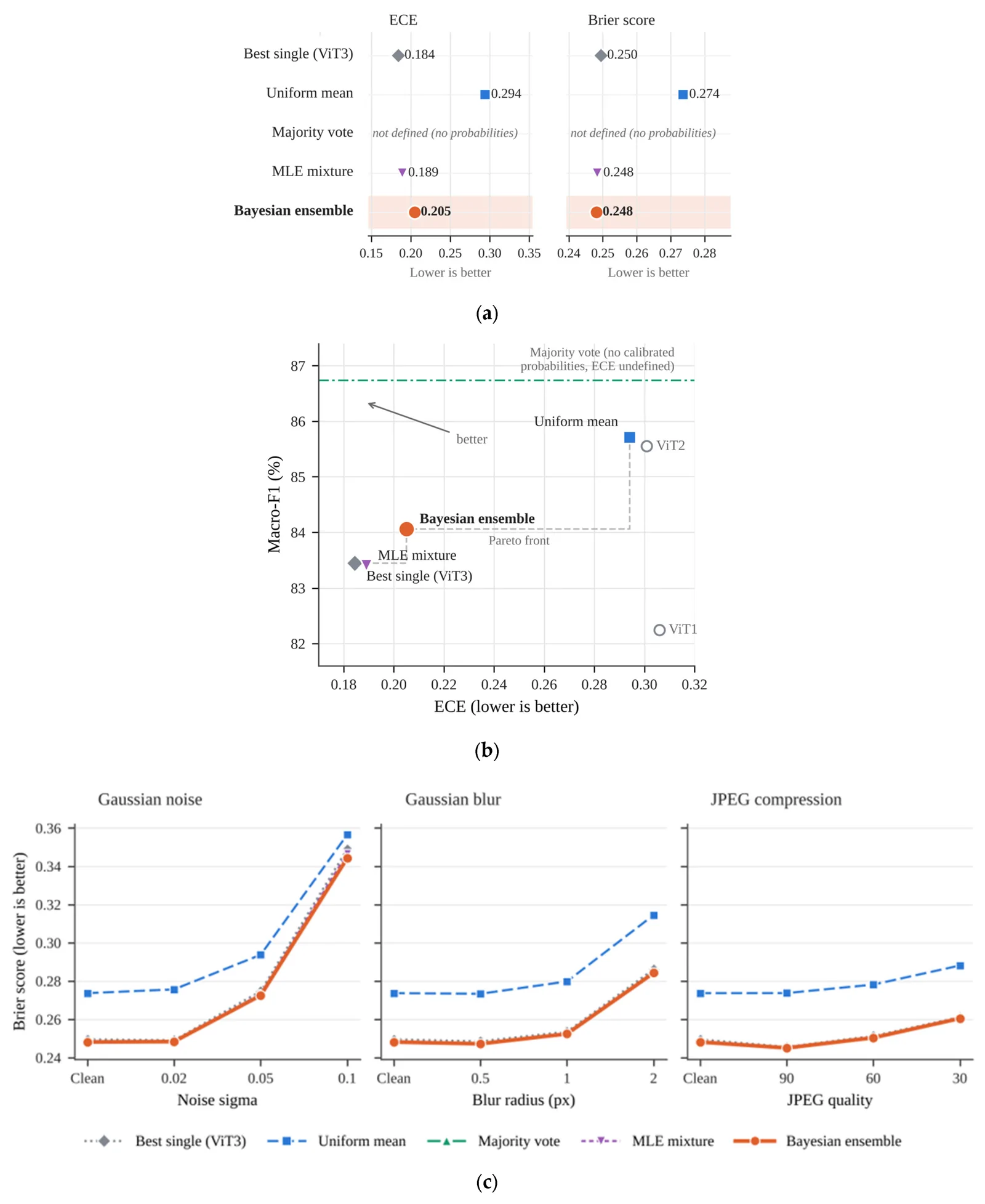
Calibration trade-off study between all models: (**a**) test performance of the best single and ensemble architectures; (**b**) ECE for the individual ViT models and the probability-based fusion rules; (**c**) Brier score under Gaussian noise, Gaussian blur, and JPEG compression.

To further assess the accuracy and robustness of the fusion models, we perform a calibration trade-off study across all models. Accuracy alone gives nothing about how far the predicted probabilities can be trusted. This matters in inspection tasks where the confidence score decides which images are sent for a manual check. Figure 12 compares ensemble fusion techniques beyond accuracy. Majority voting returns only a class label, so its calibration cannot be measured. The uniform mean ensemble returns probabilities, but they are poorly calibrated as shown in Figure 12a, with an ECE of 0.294. Compared with the uniform mean, the proposed Bayesian ensemble lowered ECE by 30% (0.205), NLL by 8.6% (0.586 against 0.641), and the Brier score by 9.3% (0.248 against 0.274). Its Brier score was the lowest of all methods, equal to that of the MLE mixture, indicating the superiority of the proposed Bayesian ensemble framework. Figure 12b shows the trade-off between accuracy and calibration. The Bayesian ensemble lies on the Pareto front, indicating that no other method has both a higher macro-F1 and a lower ECE. Moreover, moving from ViT3 to the Bayesian ensemble added 0.7 points of macro-F1. Moving further to the uniform mean added another 1.6 points of macro-F1, but it raised ECE by 0.089. Results shown in Figure 12b prove that the proposed Bayesian ensemble is the only ensemble that improves on ViT3 in macro-F1 while keeping its calibration close to that of ViT3. This can be justified by the nature of the Bayesian ensemble, which gives different weights per individual model, while the uniform mean ensemble gives each of them a fixed weight. The varied weights allow the Bayesian ensemble to learn more from the best individual model (in our case ViT3) rather than others. In terms of image disorder, Figure 12c shows that the Bayesian ensemble has the lowest Brier score in most corrupted conditions. In general, when the predicted probabilities are used for decisions, the Bayesian ensemble gives the best overall balance.

For poorly classified scaling and general defect classes, Table 10 shows the Bayesian ensemble’s class-wise performance for the general and scaling categories across five folds. Each image is evaluated once by the fold model that does not see it during training (out-of-fold, OOF). The table shows the class-wise calibration of the Bayesian ensemble before and after temperature scaling. One-vs-rest ECE uses all test images, while predicted class ECE uses only images predicted as the given class. Mean recall is 73.37% and 71.52%, while mean precision is 79.42% and 76.79%, respectively. Both tables support that calibration improves confidence estimates of both classes without eliminating all classification errors. Using temperature scaling, Table 11 shows that ECE restricted to images predicted as general or scaling falls from approximately 0.2492 and 0.2279 to 0.1528 and 0.1658.

### 4.5. Comparison with Original CNN-Based and Transformer-Based Models

**Figure 13:**
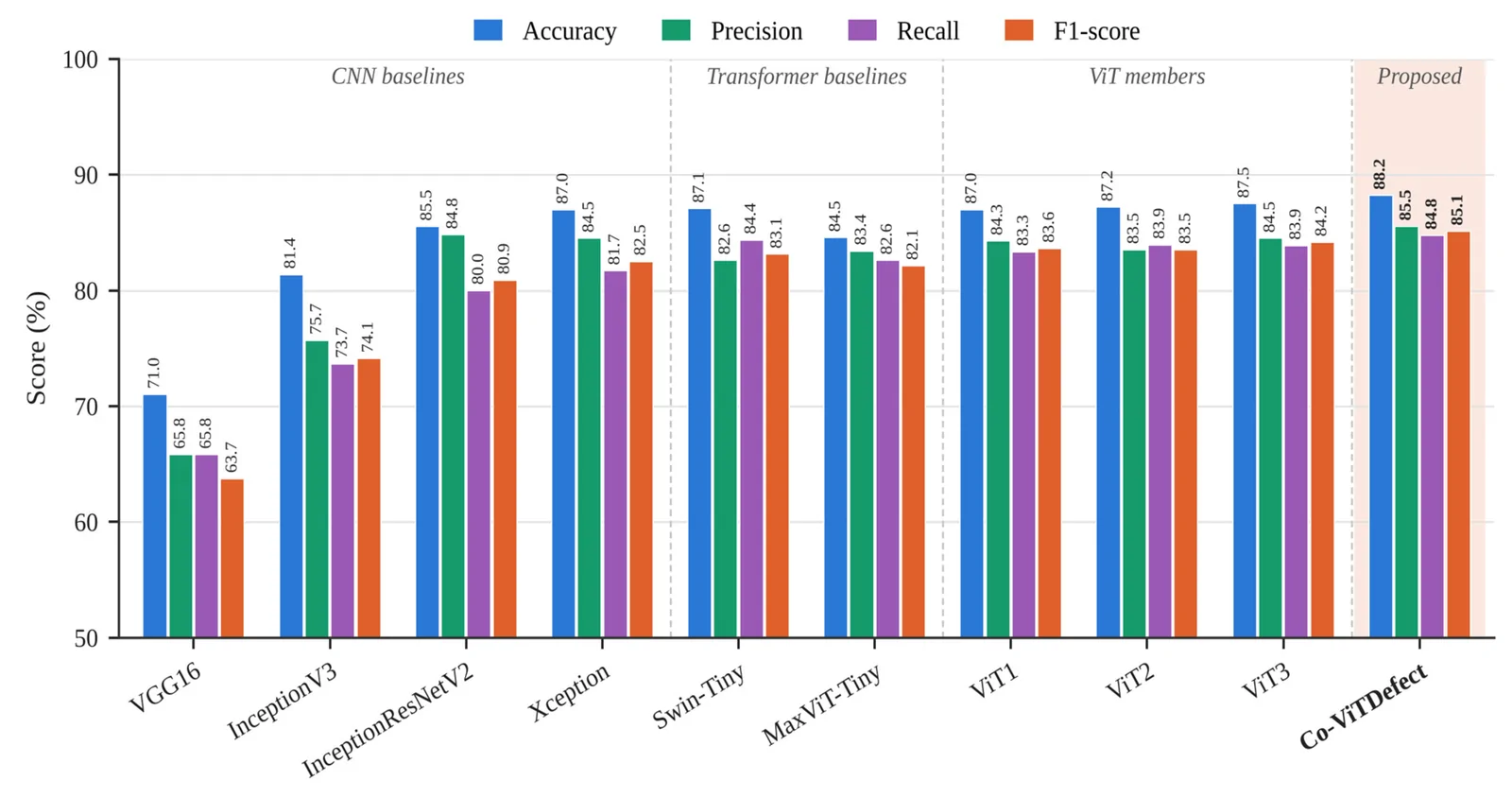
Visual comparison between CNN-based and transformer-based models with the proposed Co-ViTDefect.

To demonstrate the efficacy of the proposed Co-ViTDefect against original CNN models for multiclass concrete structure defect classification, we train several strong CNN-based models using the same dataset and training settings. Table 12 and Figure 13 provide a detailed performance comparison between Co-ViTDefect and other traditional CNN-based and transformer-based models. From the table and figure, it is observed that when comparing Co-ViTDefect against four CNN-based models (Xception, VGG16, InceptionV3, and InceptionResNetV2) using precision, recall, accuracy, and F1-score statistical metrics, Co-ViTDefect outperforms all CNN-based and transformer-based models. It achieves the best accuracy (0.8819), precision (0.8552), recall (0.8475), and F1-score (0.851), making it the most effective model for classifying defects in concrete structures compared with conventional CNN-based architectures. Xception had the highest performance among the original CNNs with an accuracy of 0.8696, precision of 0.8450, recall of 0.8169, and F1-score of 0.8251. InceptionResNetV2 (accuracy 0.8551, precision 0.8480, recall 0.8000, F1-score 0.8089) followed Xception closely, and the next best model was InceptionV3 (accuracy 0.8137, precision 0.7569, recall 0.7365, F1-score 0.7412). The model with the lowest accuracy of all evaluated models was VGG16. VGG16 had an accuracy of 0.7101, precision of 0.6581, recall of 0.6581, and F1-score of 0.6374. VGG16’s poor results reflect the limitations of its older, simpler architecture. Among transformer-based models, Swin-tiny achieved the best performance, with an accuracy of 0.8706. However, Co-ViTDefect shows strong potential and may outperform more established CNN- and transformer-based architectures in concrete structure defect classification for current and future applications, aligning with trends in the computer vision literature showing ViTs’ improved ability to model global relationships. Based on the models evaluated, Co-ViTDefect is located in the top half of the models and is recommended as the model for practice cases that require better performance in automated concrete defect detection.

**Figure 14:**
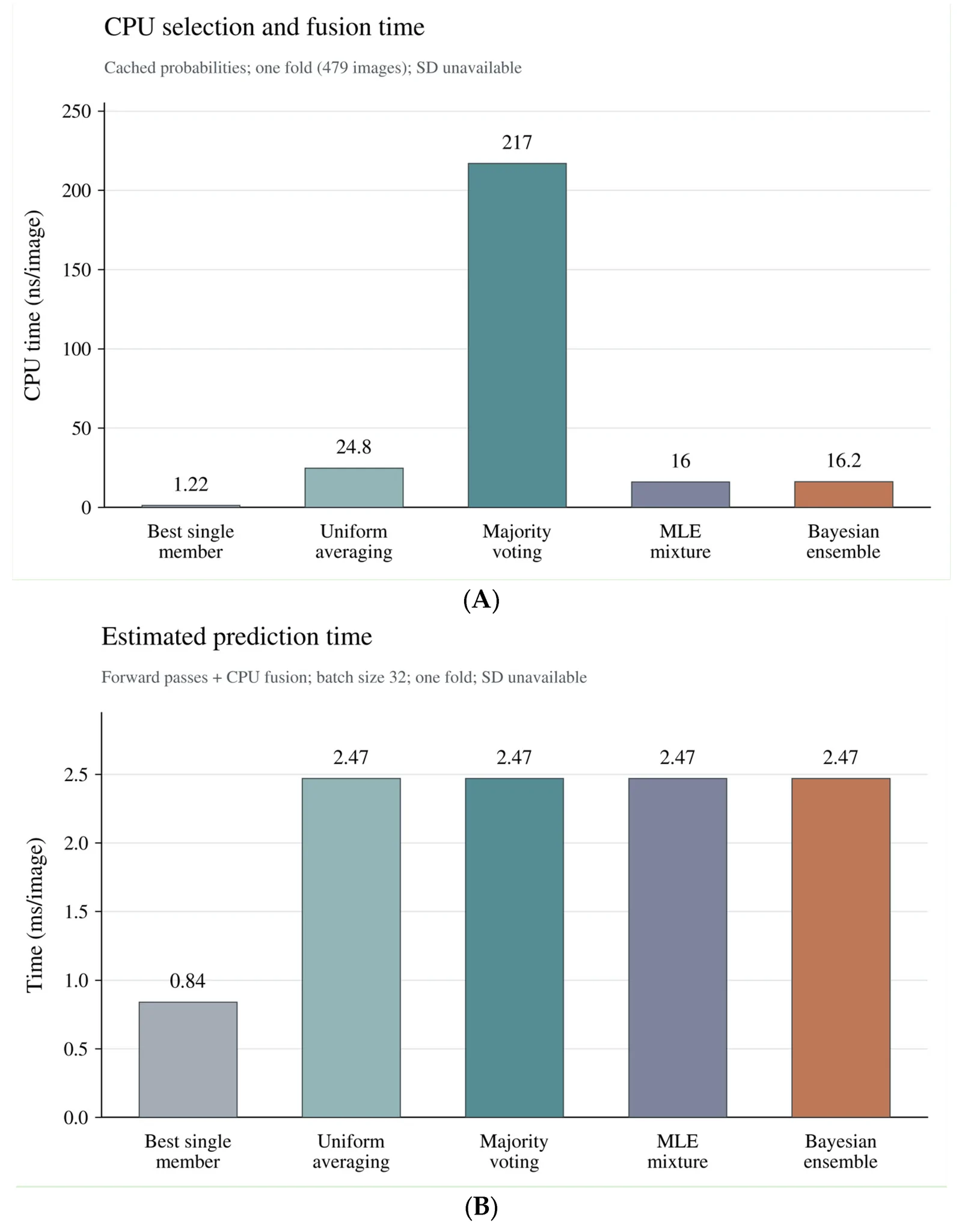
Computational time comparison of the best single ViT member and four ensemble methods: (**A**) CPU probability selection or fusion time using cached predictions; (**B**) estimated prediction time obtained by adding model-forward and CPU fusion times. Results are from one-fold containing 479 test images, with a forward-pass batch size of 32. Standard deviations are unavailable.

Computational cost was evaluated separately for the retained ViT members and fusion strategies in fold 1 (479 test images). Figure 14 summarizes the timing results. Figure 14A shows that the Bayesian ensemble required 0.0000162 ms per image for CPU fusion, close to the maximum likelihood mixture at 0.0000160 ms. Majority voting had the highest fusion time, although it contributed little to the total prediction time. As shown in Figure 14B, the best single member had an estimated prediction time of 0.84 ms per image, while all four ensemble methods averaged 2.47 ms per image. The ensemble totals were identical after rounding because the same three ViT forward passes dominated the computational cost. These results suggest that Bayesian fusion adds little prediction overhead beyond running the three members. However, the measurements cover one-fold and exclude Bayesian weight estimation, so they do not establish differences in total training cost or timing consistency across folds.

Timing used three warm-up calls and the median of seven timing repetitions. Member-forward measurements included GPU execution and transferring probabilities to the CPU but excluded image decoding, preprocessing, model loading, and compilation. CPU fusion timing excluded model execution. The single-member selection operation is not reported as fusion. Ensemble totals estimate sequential member execution and fusion, rather than directly timing a complete deployment pipeline. Posterior sampling is performed during weight fitting; prediction uses fixed posterior mean weights. These results do not establish single-image latency, five-fold timing averages, or a complete same-hardware comparison with the CNN, Swin-Tiny, and MaxViT-Tiny baselines. Bayesian weight-fitting time is also unavailable.

### 4.6. Changing the Data Augmentation Method

**Figure 15:**
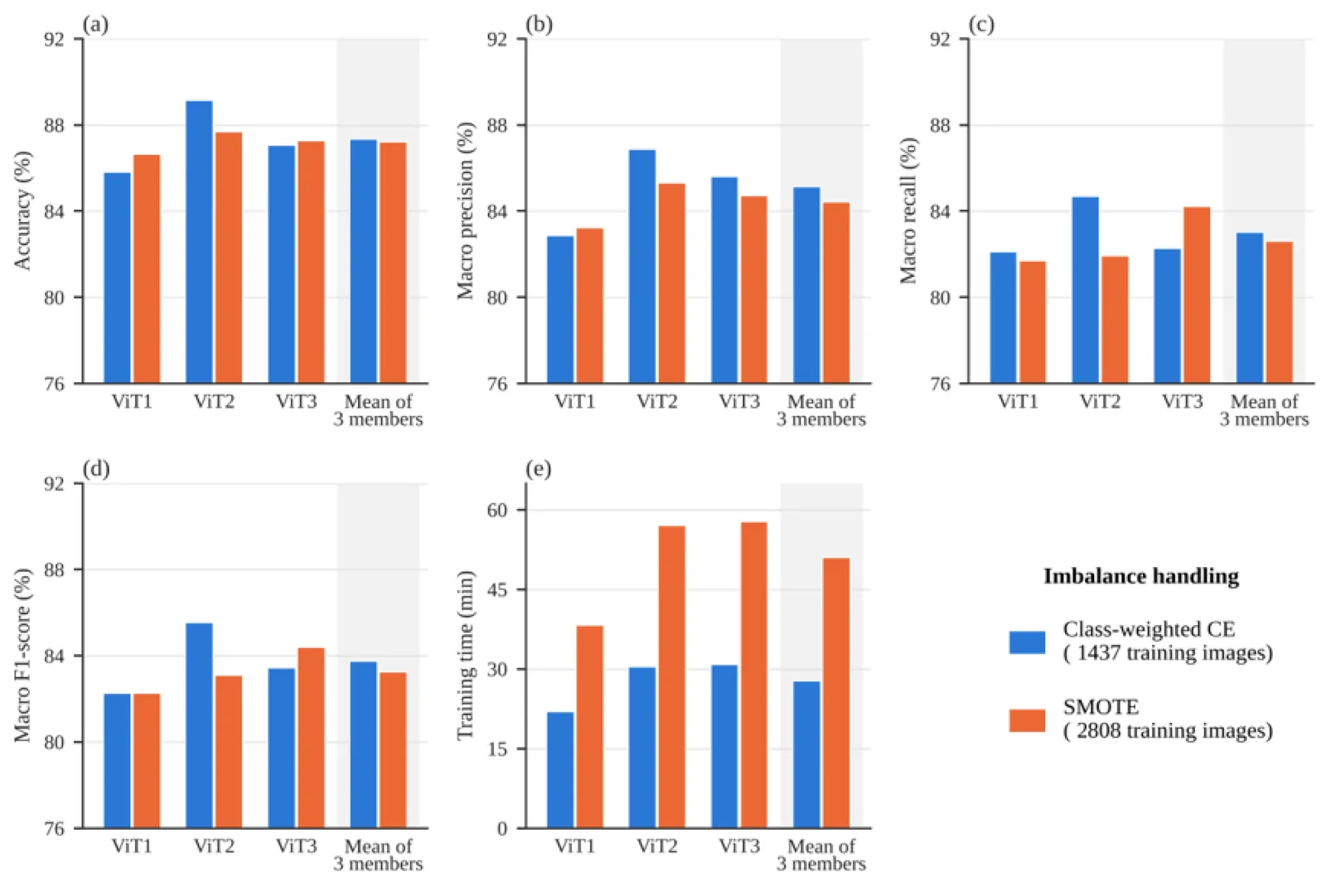
Comparison of class-weighted cross-entropy and SMOTE for the three ViT members and their average performance: (**a**) accuracy; (**b**) macro precision; (**c**) macro recall; (**d**) macro F1-score; (**e**) training time.

**Figure 16:**
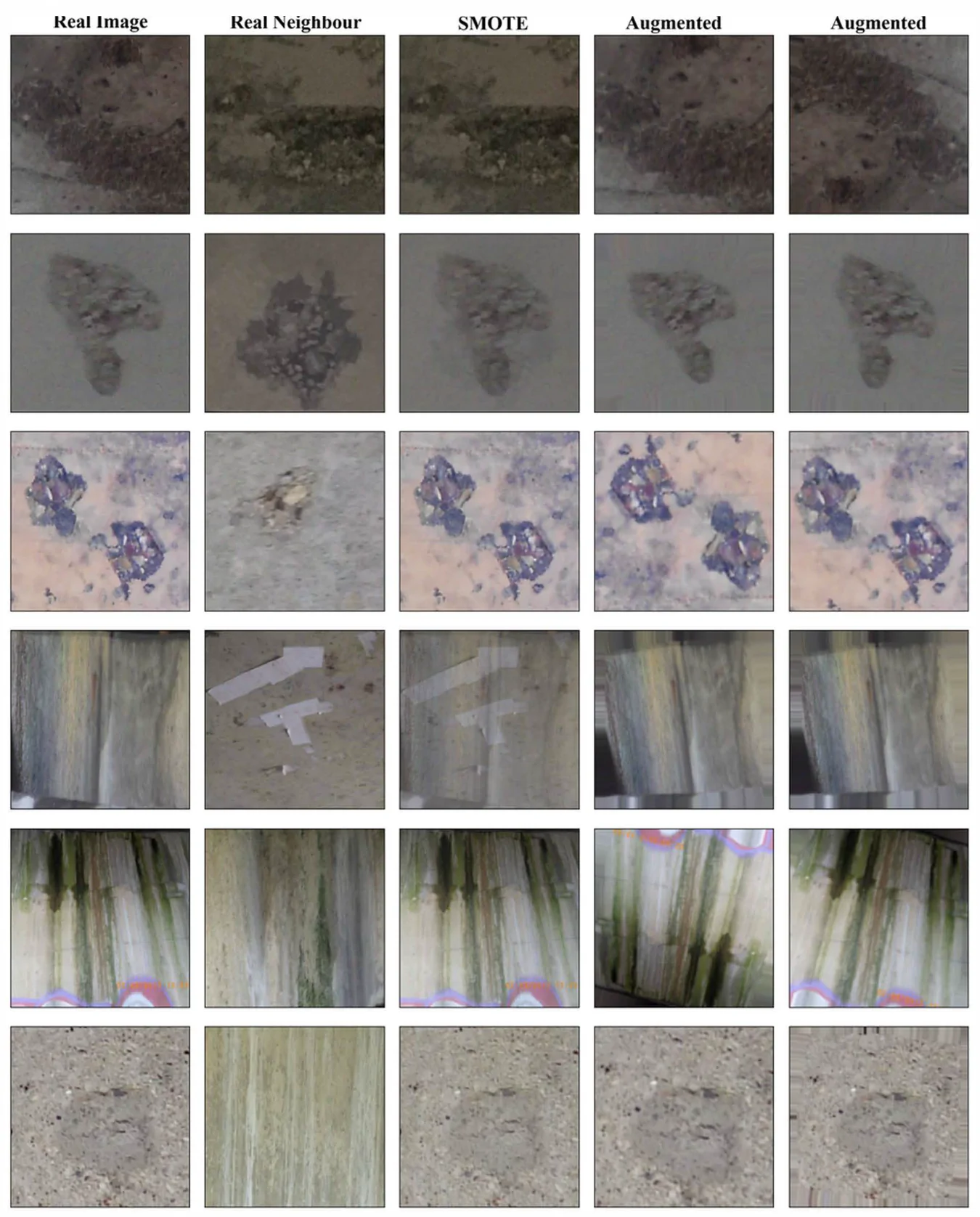
Visual comparison of data imbalance handling methods using examples from training subset.

In this section, the SMOTE algorithm is used to oversample the minor classes and balance the dataset and compare the performance with the utilized class imbalance solutions. To support this comparison, we apply SMOTE only to the training subset and separately within each class (no validation or test images are used). Each image was first resized to 224 × 224 pixels in RGB and flattened into a 150,528-dimensional vector (224 × 224 × 3). For every minority class, new samples were generated until the class had the same number of samples as the largest class. We created a new sample by choosing a random image from the class and one of its five nearest neighbours (k = 5). We rounded the resulting vectors, clipped them to the range 0 to 255, and reshaped them back into 224 × 224 × 3 images. As a result, we added 1371 synthetic images to the 1437 real training images, yielding a balanced training set of 2808 images. Figure 15 compares the performance of the individual and ensemble models using SMOTE and class-weighted methods. Figure 15 proves that SMOTE did not improve on the class-weighted loss. Averaged over the three ViT members, the class-weighted loss produced slightly higher accuracy. The mean run times were 27.72 min per member for class-weighted training and 51.04 min for SMOTE, an increase of about 84%. The corresponding ViT1, ViT2, and ViT3 times were 21.93, 30.44, and 30.80 min for class weighting and 38.26, 57.09, and 57.76 min for SMOTE. These are descriptive run-time records: the class-weighted timer also includes post-training predictions and saving, whereas the SMOTE timer primarily accumulates training loop time. They therefore do not constitute a controlled measurement of training speed with identical timing boundaries. For these reasons, the class-weighted loss was kept for the final model as the chosen class imbalance handling method. Figure 16 shows some examples of the training subset and the corresponding SMOTE and data augmentation operations. To assess degradation from image manipulation, we examined training images from Fold1 and found that both strategies produced images less sharp than the originals. Augmented images kept only 29–35% of the original sharpness, mainly because rotation and zoom resample the image and smooth fine edges. SMOTE images kept 35–53%, so by this measure they were sharper than the augmented ones.

### 4.7. External Validation

**Figure 17:**
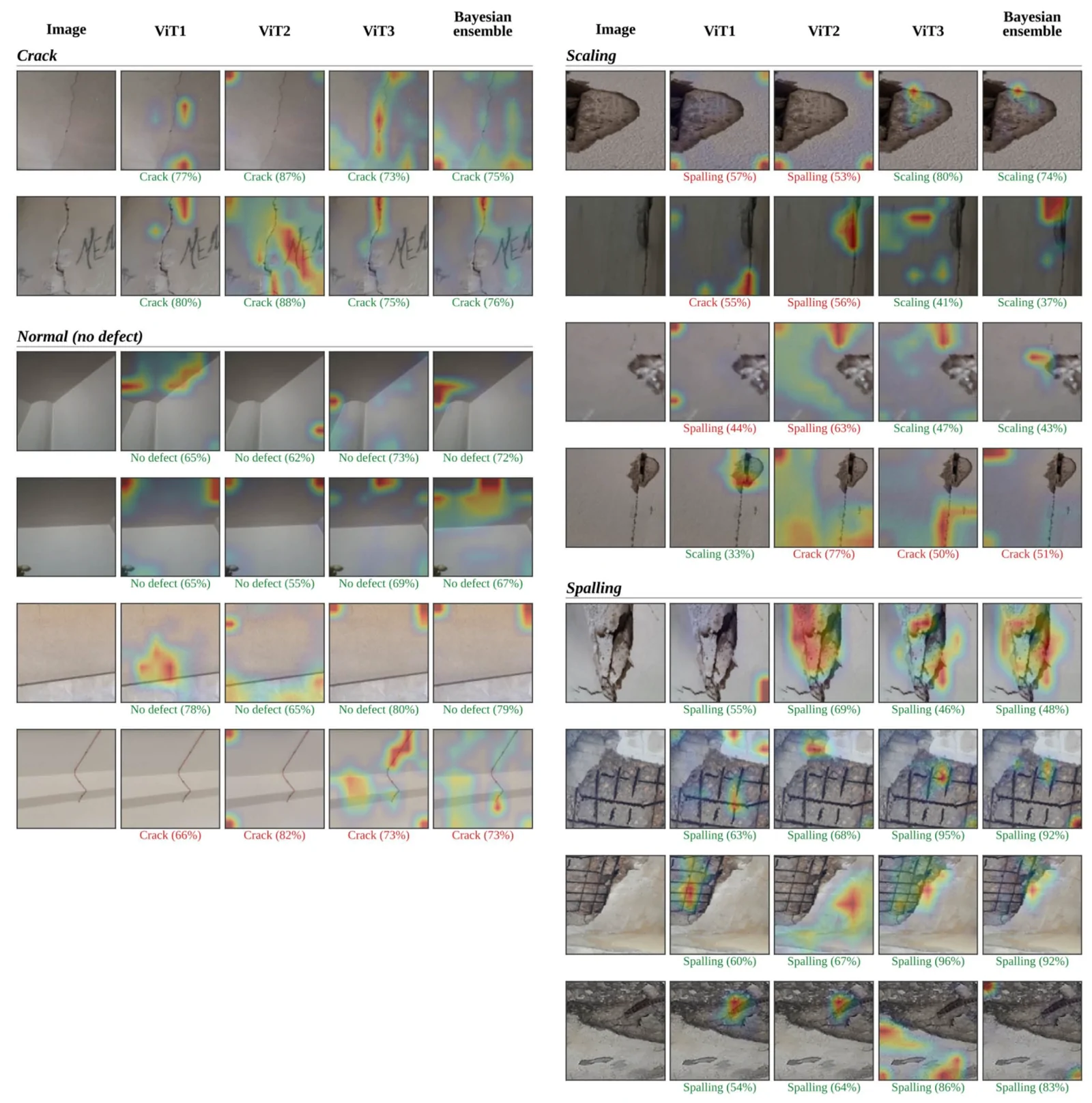
Examples of evaluating individual and ensemble models on the external data source.

To ensure generalization and the ability to implement the proposed methodology in real-life applications, an extra validation step is implemented. In this validation process, we use another open-source dataset [3,66] that contains scaling, spalling, simple cracks, severe cracks, and the normal case for concrete defects in wall and building structures (different from the dataset used). Since we do not train our models on different crack types (simple crack, severe crack), and the utilized training dataset includes two extra classes, we evaluated the trained models and the ensemble model using examples from this external dataset. This visual assessment is presented in Figure 17. The visual assessment shows that the trained ViT models and the proposed ensemble architecture can detect concrete defects across four main types (cracks, scaling, spalling, and normal). Some classification errors (mismatching) are noticed due to crack-like materials (fourth normal example) or similarity between classes like the fourth crack example, in which there is a crack and scaling in the same image; however, the annotator chose to put this sample in one category, “scaling”, which is the most obvious one. In general, the ensemble model is more capable of detecting concrete defects compared to the individual ViT models.

## 5. Conclusions

This research proposes and investigates a novel concrete structure defect detection approach called Co-ViTDefect. The discussion, comparisons, and experimental results show the developed approach’s excellent performance for multiclass defect detection, considering potential co-occurrence and scale-invariant detection. The Grad-CAM technique is utilized to further visually assess the models’ decisions in all experiments. The following remarks can be concluded:

- The developed Co-ViTDefect as an ensemble approach has improved the overall detection of the multi-defects of the individual ViT models, achieving an excellent accuracy of 88.19% after considering the difficulty of multiclass defect detection. In particular, the Co-ViTDefect approach has effectively improved the performance of the core model, particularly in the challenging “general” defect class, where Co-ViTDefect achieved an accuracy of 73.4% compared with individual accuracies of 64.3%, 66.5%, and 72.6%, respectively. In particular, Co-ViTDefect surpassed the individual ViT models, with ViT_model1, ViT_model2, and ViT_model3 registering average accuracies of 86.98%, 87.19%, and 87.48%, respectively. Based on statistical metrics, Co-ViTDefect demonstrated strong performance across most defect classes and outperformed the individual models. For the “Cracks” class, Co-ViTDefect achieved an F1-score of 0.9453 and an AUC of 0.9902, while the individual models’ F1-scores ranged from 0.934 to 0.9407 and their AUCs ranged from 0.9838 to 0.9884. Furthermore, for the “Efflorescence” class, Co-ViTDefect achieved an AUC of 0.9895, higher than the individual models. Interestingly, Co-ViTDefect significantly improves performance on the challenging “General” class, achieving an F1-score of 0.7613, compared with the individual models’ F1-scores of 0.7086 and 0.7403. Even for the “Scaling” class, Co-ViTDefect achieves an F1-score of 0.7339, competitive with the individual models. For the “Spalling” class, Co-ViTDefect maintains an F1-score of 0.8481 and an AUC of 0.9669, outperforming the best individual model (F1-score: 0.8401).
- By analyzing the patterns of misclassification, it has been revealed that the Co-ViTDefect model has reduced misclassification across most of the defect classes, which indicates its robustness for multi-defect detection. In addition, sample image analyses validate these findings, illustrating Co-ViTDefect’s ability to deliver correct predictions and avoid individual model misclassifications. However, the difficulty of accurately classifying “Scaling” defects is supported by its highest misclassification rates and lowest performance scores across all models. This warrants further investigation.
- In comparison with other ensemble techniques for Co-ViTDefect with Dirichlet weighted ensemble, the accuracies of the other ViT ensembles are also good. Uniform averaging and majority voting gave the highest accuracies compared with other ensemble strategies (e.g., maximum likelihood mixture achieved a lower accuracy of 87.9%). However, accuracy is not the only factor when the model is used for inception decisions. Majority voting returns only a class label with no probability, and uniform averaging produced poorly calibrated probabilities, with an ECE of 0.285 against 0.214 for the Bayesian ensemble. The Bayesian ensemble also had the lowest raw Brier score (0.242). This balanced performance suggests that Co-ViTDefect has more stable, consistent classification across a broad range of defect types in concrete structure inspections. Other ensemble methods, even when they achieve comparable overall accuracy, may be less consistent across defect classes.
- Co-ViTDefect has exhibited superior performance with lower rates of misclassification compared with traditional CNN-based and other advanced transformer-based models, particularly among structurally disparate defect types. Most other errors can be attributed to visually overlapping or low-prevalence classes, including the “Scaling” and “General” classes, regardless of the algorithm type, and these classes will consistently challenge classification algorithms. This indicates that even with meticulous model design, some misclassification will still occur due to limited data diversity and annotation granularity. Future work should include multi-label classification, region-level annotation, and richer datasets to address these limitations further.
- Validating the proposed Co-ViTDefect ensemble using an extra concrete defect dataset proved the robustness and reliability of the proposed framework.
- Future work should also address several areas of improvement. First, the “Scaling” class detection should benefit from further evaluation and improvement, which can be achieved by addressing the problem of unbalanced data, as well as refining the ViT model architecture and ensemble learning technique. Moreover, during actual inspections, we may encounter defects with overlapping characteristics or indistinct boundaries (e.g., combined cracking and scaling). Future work should evaluate multi-label classification or instance segmentation methods to handle unknown or compound defects more accurately. Lastly, the multiclassification framework developed in Co-ViTDefect could be further integrated with other evaluation tasks, such as region-based detection and pixel-level classification, to improve accuracy, narrow the area of interest for defects, and support accurate localization and severity assessment.

## Figures and Tables

**Table 1:** Dataset specification.

Class	Files in Main Dataset Archive	Conflicting-Label Excluded Files	Duplicated Removed Files	Unique Images Used
Cracks	789	5	4	780
Efflorescence	311	5	0	306
General defect	264	0	1	263
No defect	452	0	0	452
Scaling	168	0	0	168
Spalling	427	0	0	427
Total	2411	10	5	2396

**Table 2:** Distribution of images across the training, validation, calibration, and test subsets for one representative cross-validation fold.

Class	Train	Validation	Calibration	Test
Cracks	468	78	78	156
Efflorescence	184	30	31	61
General defect	158	26	26	53
No defect	271	46	45	90
Scaling	100	17	17	34
Spalling	256	43	43	85
Total	1437	240	240	479

**Table 3:** Individual-model performance from pooled out-of-fold predictions for 2396 images. Values are proportions. AUC is calculated one-versus-rest; the macro AUC is the unweighted mean of the six class AUCs.

Model	Class	Precision	Recall	F1-Score	AUC	Accuracy
ViT1	Cracks	0.9246	0.9436	0.9340	0.9838	0.8698
	Efflorescence	0.8555	0.9477	0.8992	0.9837	
	General defect	0.7897	0.6426	0.7086	0.9153	
	No defect	0.9161	0.8938	0.9048	0.9792	
	Scaling	0.7516	0.7202	0.7356	0.9520	
	Spalling	0.8180	0.8525	0.8349	0.9517	
	Macro avg	0.8426	0.8334	0.8362	0.9610	
	Weighted avg	0.8682	0.8698	0.8677	-	
ViT2	Cracks	0.9298	0.9513	0.9404	0.9884	0.8719
	Efflorescence	0.8997	0.9379	0.9184	0.9884	
	General defect	0.7778	0.6654	0.7172	0.9139	
	No defect	0.9273	0.9027	0.9148	0.9768	
	Scaling	0.6184	0.7619	0.6827	0.9348	
	Spalling	0.8575	0.8173	0.8369	0.9517	
	Macro avg	0.8351	0.8394	0.8351	0.9590	
	Weighted avg	0.8741	0.8719	0.8718	-	
ViT3	Cracks	0.9365	0.9449	0.9407	0.9871	0.8748
	Efflorescence	0.9065	0.9183	0.9123	0.9882	
	General defect	0.7549	0.7262	0.7403	0.9388	
	No defect	0.8954	0.9093	0.9023	0.9834	
	Scaling	0.7389	0.6905	0.7138	0.9256	
	Spalling	0.8372	0.8431	0.8401	0.9626	
	Macro avg	0.8449	0.8387	0.8416	0.9643	
	Weighted avg	0.8734	0.8748	0.8740	-	

**Table 4:** Pairwise test-error diversity across five folds, mean ± SD.

Member Pair	Prediction Disagreement (%)	Both Wrong (%)	Error Correlation	Q Statistic
ViT1–ViT2	10.56 ± 1.13	8.56 ± 1.94	0.608 ± 0.074	0.947 ± 0.019
ViT1–ViT3	11.19 ± 1.45	8.31 ± 1.24	0.601 ± 0.058	0.945 ± 0.020
ViT2–ViT3	11.06 ± 1.60	8.14 ± 1.18	0.592 ± 0.080	0.939 ± 0.031

**Table 5:** Attention maps and class activation maps for some examples for all trained ViT models.

Original Image	ViTmodel1	ViTmodel2	ViTmodel3
	Attention Map		
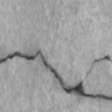	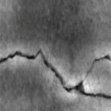	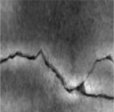	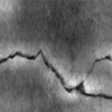
	ViT Grad-CAM		
	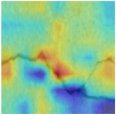	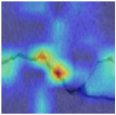	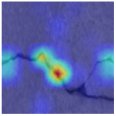
Original Image	Attention Map		
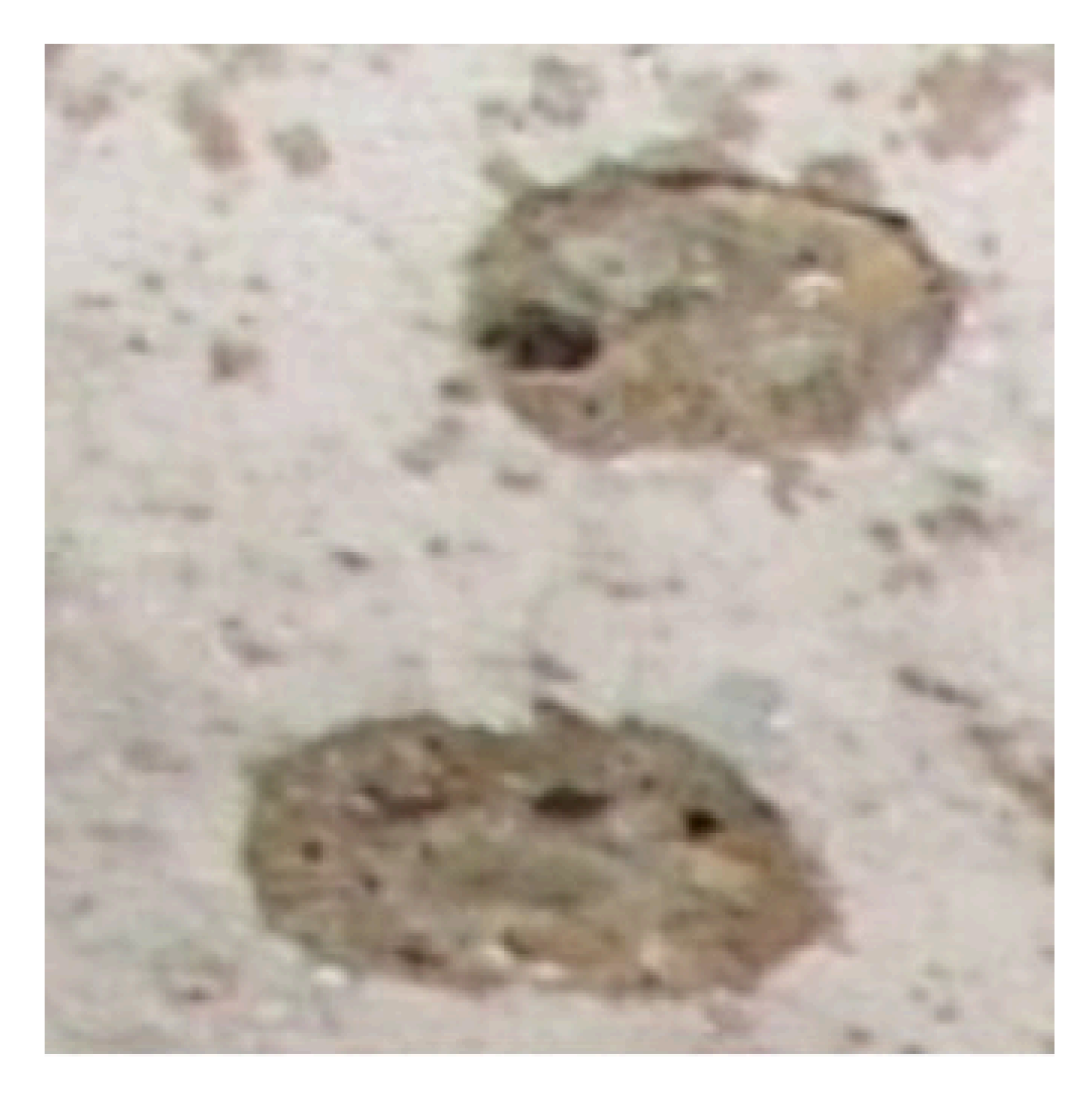	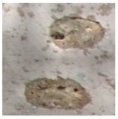	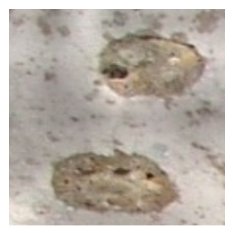	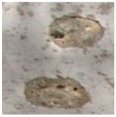
	ViT Grad-CAM		
	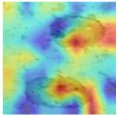	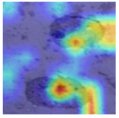	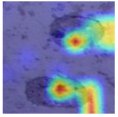
Original Image	Attention Map		
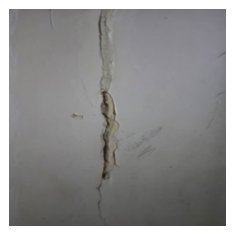	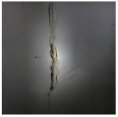	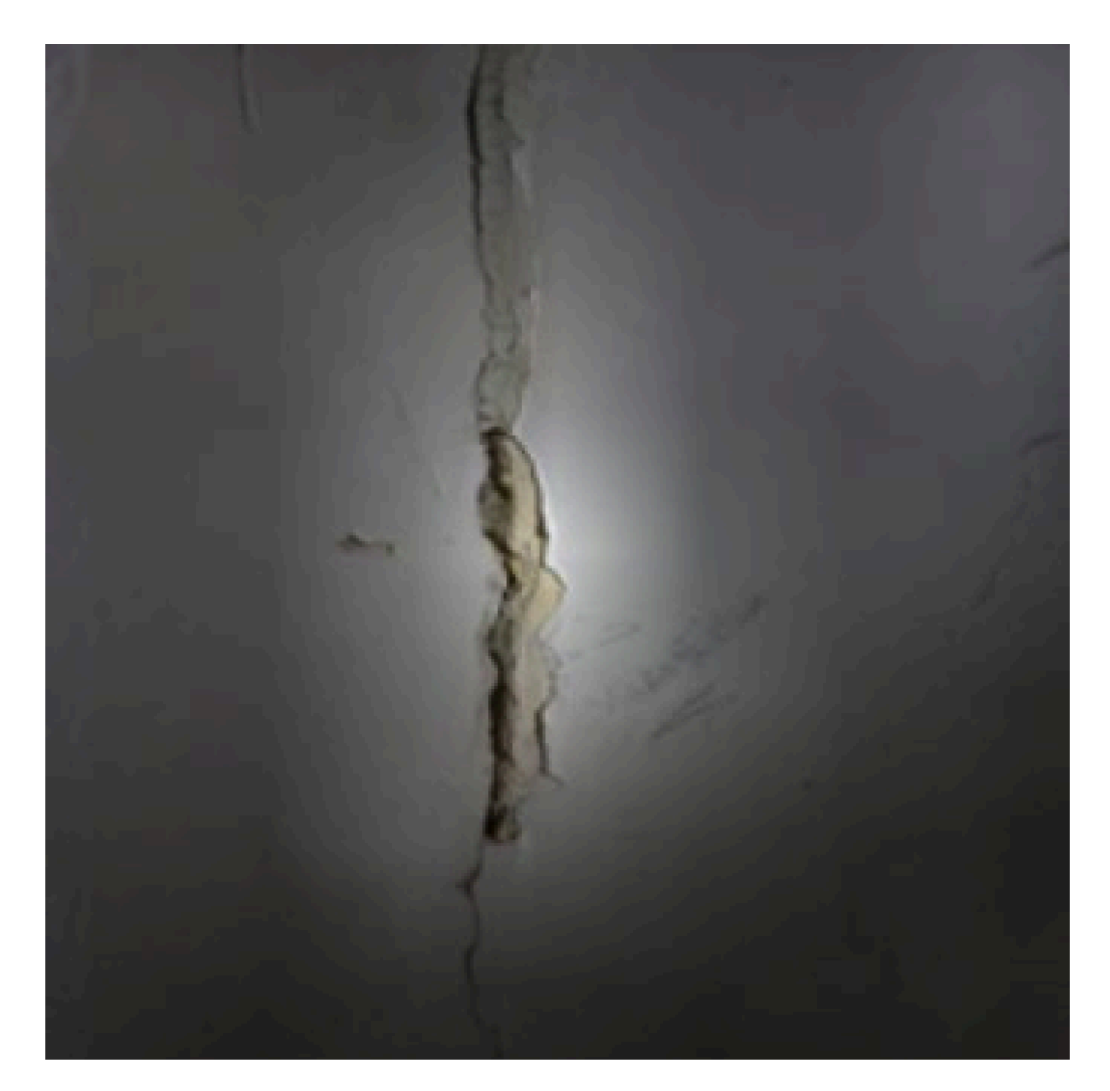	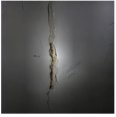
	ViT Grad-CAM		
	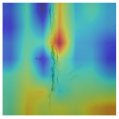	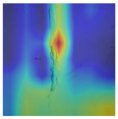	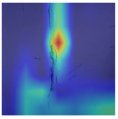
Original Image	Attention Map		
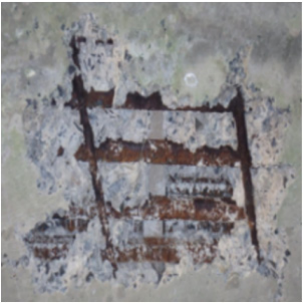	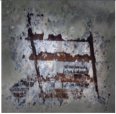	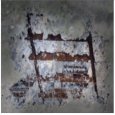	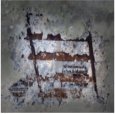
	ViT Grad-CAM		
	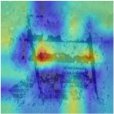	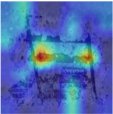	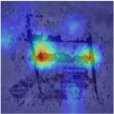

**Table 6:** Bayesian Co-ViTDefect performance from pooled out-of-fold predictions for 2396 images. Values are proportions; AUC is calculated one-versus-rest.

Co-ViTDefect	Precision	Recall	F1-Score	AUC	Accuracy
Cracks	0.9346	0.9526	0.9435	0.9902	0.8819
Efflorescence	0.9013	0.9248	0.9129	0.9895	
General defect	0.7910	0.7338	0.7613	0.9450	
No defect	0.9033	0.9093	0.9063	0.9865	
Scaling	0.7547	0.7143	0.7339	0.9386	
Spalling	0.8462	0.8501	0.8481	0.9669	
Macro avg	0.8552	0.8475	0.8510	0.9694	
Weighted avg	0.8803	0.8819	0.8809	-	

**Table 7:** Test performance across the same five folds (mean ± standard deviation) for individual and ensemble models.

Model	Accuracy (%)	Macro F1 (%)
ViT1	86.98 ± 2.31	83.62 ± 3.30
ViT2	87.19 ± 1.56	83.51 ± 2.17
ViT3	87.48 ± 0.89	84.18 ± 1.12
Bayesian ensemble	88.19 ± 1.41	85.12 ± 1.85

**Table 8:** Test performance across the same five folds (mean ± standard deviation) for different ensemble strategies.

Model	Accuracy (%)	Macro F1 (%)
Uniform averaging	89.57 ± 1.33	86.72 ± 2.27
Majority voting	89.31 ± 1.39	86.42 ± 2.13
Maximum likelihood mixture	87.90 ± 1.31	84.66 ± 1.67
Bayesian ensemble	88.19 ± 1.41	85.12 ± 1.85

**Table 9:** Calibration measures across the five held-out test folds, reported as mean (standard deviation). Raw denotes probabilities before temperature scaling; calibrated denotes probabilities after temperature scaling.

Method	NLL	Brier Score	ECE
ViT3, raw	0.5680 ± 0.0450	0.244 (0.023)	0.190 (0.049)
ViT3, calibrated	0.4411 ± 0.0334	0.1931 (0.0167)	0.045 (0.011)
Uniform, raw	0.6217 ± 0.0287	0.2642 ± 0.0142	0.285 (0.040)
Uniform, calibrated	0.3589 ± 0.0342	0.1595 ± 0.0178	0.036 (0.008)
Bayesian, raw	0.5698 ± 0.0415	0.2422 ± 0.0207	0.214 (0.051)
Bayesian, calibrated	0.4030 ± 0.0425	0.1802 ± 0.0203	0.042 (0.010)

**Table 10:** Class-wise performance of the Bayesian ensemble for the general and scaling classes across five folds, mean ± SD.

Class	Total OOF Support	Precision (%)	Recall (%)	F1 (%)
General	263	79.42 ± 4.78	73.37 ± 4.87	76.13 ± 2.87
Scaling	168	76.79 ± 11.29	71.52 ± 7.38	73.60 ± 6.65

**Table 11:** Class-wise calibration of the Bayesian ensemble before (raw) and after temperature scaling, mean ± SD.

Class	Probabilities	One-vs-Rest ECE	Predicted Class ECE
General	Raw	0.0632 ± 0.0124	0.2492 ± 0.0812
General	Calibrated	0.0279 ± 0.0059	0.1528 ± 0.0278
Scaling	Raw	0.0683 ± 0.0153	0.2279 ± 0.0521
Scaling	Calibrated	0.0285 ± 0.0075	0.1658 ± 0.0796

**Table 12:** Comparison between CNN-based and transformer-based models with the proposed Co-ViTDefect.

Model	Accuracy	Precision	Recall	F1-Score
ViT1	0.8698	0.8426	0.8334	0.8362
ViT2	0.8719	0.8351	0.8394	0.8351
ViT3	0.8748	0.8449	0.8387	0.8416
Xception	0.8696	0.8450	0.8169	0.8251
VGG16	0.7101	0.6581	0.6581	0.6374
InceptionV3	0.8137	0.7569	0.7365	0.7412
InceptionResNetV2	0.8551	0.8480	0.8000	0.8089
Swin-Tiny	0.8706	0.8259	0.8436	0.8312
MaxViT-Tiny	0.8455	0.8340	0.8259	0.8212
**Co-ViTDefect**	0.8819	0.8552	0.8475	0.8510

## Data Availability

The data used in this paper is publicly available using the following repository: https://zenodo.org/records/2601506 (accessed on 15 October 2026).

## Abbreviations

CNN, convolutional neural network; ViT, vision transformer; SHM, structural health monitoring; DEL, Dirichlet ensemble learning; MLP, multilayer perceptron; CAM, class activation map; Grad-CAM, gradient-weighted class activation mapping; ROC, receiver operating characteristic; AUC, area under the receiver operating characteristic curve; NLL, negative log-likelihood; ECE, expected calibration error; SD, standard deviation; OOF, out-of-fold; SMOTE, synthetic minority oversampling technique.
